# Synthesis and multilevel evaluation of benzimidazole-based anticancer compounds: DNA/serum albumin interaction, and in-depth theoretical insights

**DOI:** 10.1007/s11010-026-05600-3

**Published:** 2026-06-23

**Authors:** Sevgi Kundu, Senem Akkoc, Yalçın Erzurumlu, Sadeq K. Alhag, Lamya Ahmed Alkeridis, Mehran Feizi-Dehnayebi

**Affiliations:** 1 Institute of Health Sciences, Department of Basic Pharmaceutical Sciences, Suleyman Demirel University, Isparta, 32260 Türkiye Turkey; 2 Faculty of Pharmacy, Department of Basic Pharmaceutical Sciences, Suleyman Demirel University, Isparta, 32260 Türkiye Turkey; 3 Faculty of Engineering and Natural Sciences, BahçeşehirUniversity, Istanbul, 34349 Türkiye Turkey; 4 Faculty of Pharmacy, Department of Biochemistry, Suleyman Demirel University, 32260 Isparta, Turkey; 5 Health Specialties, Basic Sciences and Applications Unit, King Khalid University, Applied College, Mohayil Asir Abha, 61421 Saudi Arabia; 6 Department of Biology, Collage of Science, Princess Nourah bint Abdulrahman University, P.O.Box 84428, Riyadh, 11671 Saudi Arabia

**Keywords:** Cancer, Benzimidazole, Cytotoxic activity, Apoptosis, Oxidative stress, DNA/BSA, Theoretical studies

## Abstract

Within the scope of this investigation, two novel compounds (**3a** and **3b**) were designed and synthesized in two steps. Compounds **3a** and **3b** were tested utilizing the MTT study to evaluate their in vitro cytotoxic activity against healthy human embryonic kidney, lung cancer, breast cancer, and human liver cancer cell lines. It was determined that compound **3b** exhibited high levels of cytotoxic activity against both liver and breast cancer cell lines, with IC_50_ values of 10.83 and 11.55 µM, respectively. Also, to elucidate the anticancer mechanism of compounds, pro-apoptotic *BAX* and *BiD*, anti-apoptotic *BCL2* and *BCL-xl*, oxidant enzymes *PRDX1* and *SOD1* levels were examined by RT-qPCR. Moreover, cellular oxidative stress levels were spectrophotometrically measured, and cellular senescence was evaluated via the senescence-associated β-galactosidase test. DFT calculations and RDG, ELF, and LOL analyses were performed to demonstrate the reactivity of these compounds. Compounds significantly down-regulated anti-apoptotic genes, whereas mRNA levels of pro-apoptotic genes were up-regulated in MCF-7 cells. Moreover, oxidative stress status was significantly increased depending on the compound treatment. Consistently, PRDX1 and SOD1 levels were also significantly up-regulated. Furthermore, cellular senescence was significantly induced by the compounds. Fluorescence spectroscopy demonstrated strong binding of both compounds with CT-DNA, characterized by static quenching mechanism and binding constants of 6.02 × 10^6^M^− 1^(for **3a**) and 8.69 × 10^6^M^− 1^(for **3b**), suggesting an intercalative binding mode. In contrast, moderate affinity toward BSA indicated suitable transport characteristics with reduced nonspecific protein binding. Molecular docking studies supported the experimental findings. These combined results verify the potential of the synthesized derivatives as promising candidates for further investigation as biologically active anticancer agents.

## Introduction

The rapid increase in cancer cases worldwide and the parallel rise in mortality rates have made cancer a serious global health problem. The development of resistance to treatment, the toxic effects of the drugs used, the risk of recurrence, and the low success rate of drugs reaching the clinical stage are among the fundamental challenges in cancer treatment [1]. Breast cancer, the leading malignancy diagnosed in women globally, affects over two million individuals each year, posing a major public health challenge [2].

Among organic compounds, heterocyclic systems hold an important place in medicinal chemistry [3]. Heterocyclic compounds constitute an important pharmacophore structure in the development of new drugs due to their antibacterial, anticancer, antiviral, and various biological activity properties [4–7]. Nitrogen atoms are critical for the biological activity of these compounds, as they influence the capacity of heterocyclic structures to interact with DNA, proteins, and enzymes [8].

Benzimidazole, one of the oldest known nitrogen-containing heterocyclic compounds, is formed through the fusion of the benzene ring with the 4th and 5th positions of the imidazole ring [9]. The use of imidazole and benzimidazole derivatives containing nitrogen heteroatoms in medicinal chemistry has shown significant developments in recent years. These compounds are found in the structure of many current drug active ingredients and contribute significantly to the therapeutic management of various diseases. In addition, intensive research is ongoing on a global scale on new imidazole and benzimidazole derivatives that are being evaluated for their therapeutic potential [10]. Benzimidazole derivatives have been studied for their anticancer [11–13], antituberculosis [14, 15], antibacterial [16–18], antioxidant [19], acetylcholinesterase inhibitor [20], and anti-inflammatory [21] activities, which have been investigated by different researchers.

**Fig. 1:**
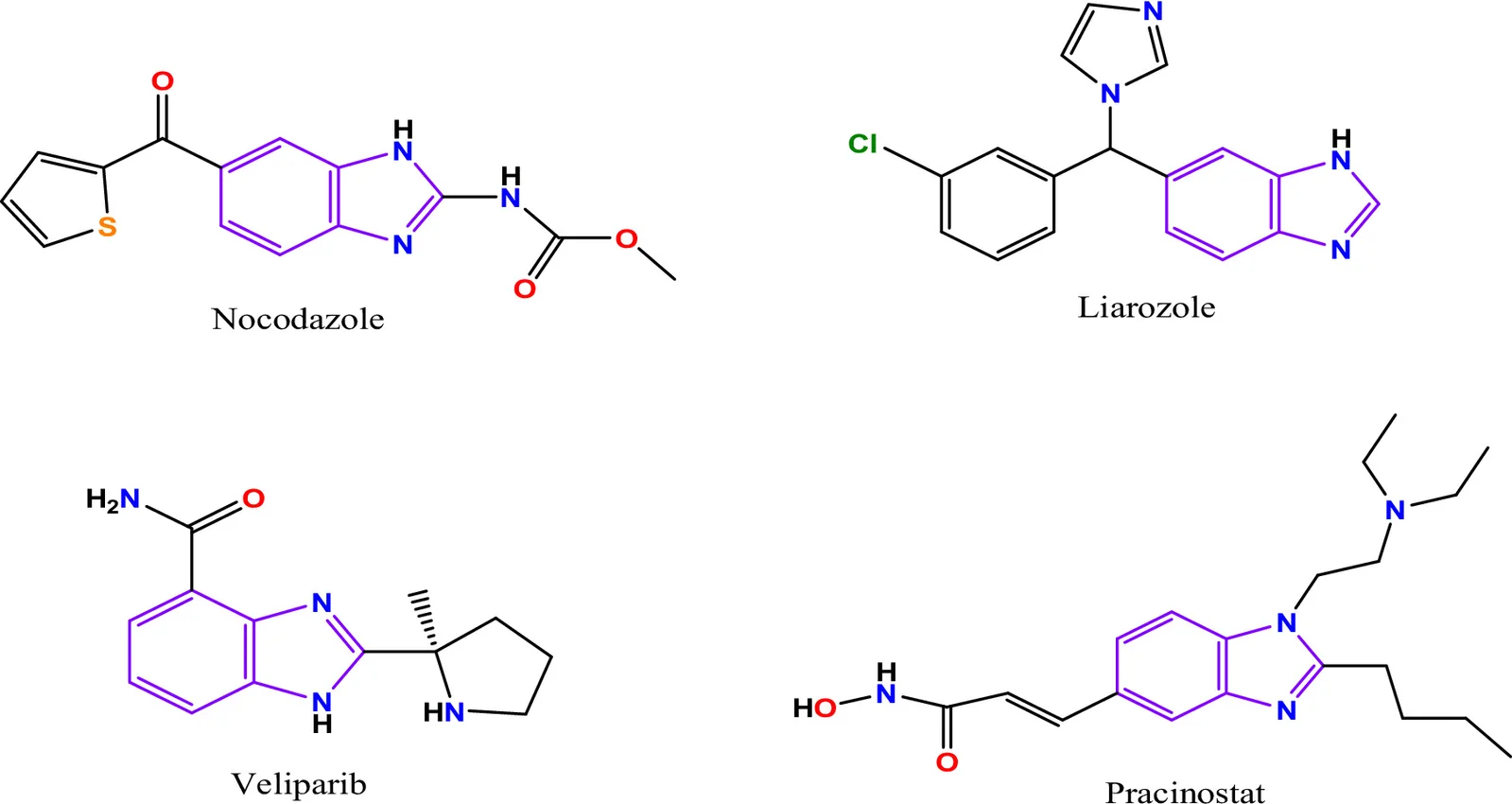
Some antineoplastic agents containing a benzimidazole core

Among the anticancer drugs developed in recent years, benzimidazole derivatives have come to the fore due to their various biological effects and therapeutic potential. The unique ring structure and low toxicity of these compounds make them a valuable building block in the design of new-generation anticancer drugs [1]. Nocodazole, liarozole, veliparib, and pracinostat (Fig. 1) are benzimidazole derivative antineoplastic agents undergoing different clinical phase studies. These derivatives can bind to tubulin and inhibit microtubule formation, thereby causing cancer cell death [22]. Benzimidazole derivatives have been studied in leukemia [23], breast [24], lung [25], liver [26], colorectal [27], and prostate cancers [28].

Numerous anticancer molecules reported in the literature are known to induce oxidative stress by disrupting the redox balance in cancer cells, thereby activating proliferation-limiting mechanisms such as cellular senescence. These changes occurring can also trigger programmed cell death processes in cancer cells [29] At this point, the shift in the balance between pro-apoptotic and anti-apoptotic genes, such as *BAX*, *BiD*, *BCL2* and *BCL-xL*, and the signaling pathways induced at the protein level play a critical role in determining the ultimate fate of cells [30]. Targeting these regulations constitutes one of the fundamental approaches in the development of anticancer strategies. Thus, evaluating the mechanistic effects of developed potent anticancer agents is important for a more accurate and comprehensive interpretation of the results obtained at the cellular level by including these processes [31].

In our previous study, various benzimidazole derivatives were synthesized and their cytotoxic activities were investigated against A549, HepG2, and MCF7 cell lines [32]. Furthermore, DFT calculations and molecular docking studies against cancer-related protein (PDB ID: 3ERT), DNA (PDB ID: 1Z3F), and BSA (PDB ID: 4JK4) were performed for **3a** and **3b**. Based on these findings, new benzimidazolium salts were synthesized and their biological activities were investigated in the present work.

## Experimental section

### Synthesis of compounds

*Preparation of 1-(3-chlorobenzyl)−2-methyl-1 H-benzo[d]imidazole (2)*

Potassium hydroxide (0.23 g) was added to a solution of compound 1 (0.5 g) in ethanol (4 mL), and the mixture was stirred at room temperature. 1-Chloro-3-(chloromethyl)benzene (479.70 µL) was then introduced, and the reaction progress was monitored by TLC. Upon completion, the reaction mixture was filtered.

*Synthesis of 1-(3-chlorobenzyl)−3-(3-methylbenzyl)−2-methyl-benzimidazolium chloride (3a)*.

Compound **2** (0.3 g) was dissolved in chloroform (2 mL), and 1-(chloromethyl)−3-methylbenzene (154.43 µL) was added. The reaction mixture was refluxed at 60 °C, and progress was monitored by TLC. Upon completion, the mixture was filtered, and the filtrate was allowed to crystallize. The resulting white solid was recrystallized from isopropyl alcohol, washed with diethyl ether, and dried. Yield: 60%, m.p: 254–259 °C, color: white. ^1^H NMR (400.13 MHz, DMSO-d_6_, 298 K), δ: 2.28 and 3.01 (s, 6 H, 2 x C*H*_3_), 5.75 and 5.87 (s, 4 H, 2 x C*H*_2_), 7.15–7.97 (m, 12 H, Ar-*H*). ^13^C NMR (100 MHz, DMSO-d_6_, 298 K), δ: 11.64 and 21.41 (*C*H_3_); 47.32 and 48.17 (*C*H_2_); 113.11, 113.86, 114.77, 124.87, 125.86, 126.89, 127.73, 128.74, 128.85, 129.35, 131.32, 131.61, 132.34, 134.07, 134.48, 137.37, 138.81, 152.50, and 153.51 (Ar-*C*; N*C*N).

*Synthesis of 1-(3-chlorobenzyl)−3-(4-methylbenzyl)−2-methyl-benzimidazolium chloride (3b)*

Compound **2** (0.3 g) was dissolved at room temperature with stirring in a chloroform/ethanol mixture (2:1, mL). 1-(Chloromethyl)−4-methylbenzene (154.43 µL) was then added to the reaction mixture. The reaction was refluxed at 70 °C, and its progress was monitored by TLC. After completion, the mixture was filtered, and the filtrate was allowed to stand for crystallization, during which gradual precipitation of a solid was observed. The solid substance was crystallized with isopropyl alcohol. It was observed that the solid substance gradually precipitated in the crystallized filtrate. The solid substance was filtered off from the liquid portion. The substance washed with diethyl ether was left to dry. Yield: 70%, m.p: 263.5–267 °C, color: white. ^1^H NMR (400.13 MHz, DMSO-d_6_, 298 K), δ: 2.28 and 3.01 (s, 6 H, 2 x C*H*_3_), 5.78 and 5.85 (s, 4 H, 2 x C*H*_2_), 7.18–7.96 (m, 12 H, Ar-*H*). ^13^C NMR (100 MHz, DMSO-d_6_, 298 K), δ: 11.60 and 21.12 (*C*H_3_); 48.14 and 48.71 (*C*H_2_); 113.67, 113.73, 113.90, 126.45, 126.88, 127.69, 127.73, 127.92, 128.84, 129.97, 131.29, 131.33, 131.44, 131.56, 134.56, 137.01, 137.65, 138.26, and 153.12 (Ar-*C*; N*C*N). LC-MS/MS = m/z: [M-Cl]^+^ for C_23_H_22_N_2_Cl calculated: 361.90 g/mol; found: 361.05 g/mol.

### Cytotoxic activity studies

HEK-293T, A549, and HepG2 cells were cultured in DMEM supplemented with 10% FBS and 1% GlutaMAX, while MCF-7 cells were maintained in RPMI instead of DMEM. Cells were seeded in sterile 96-well plates at a density of 4 × 10^3^ cells per well and incubated for 24 h at 37 °C in a humidified atmosphere with 5% CO_2_. Following this, cells were treated with the test compounds at concentrations ranging from 9.375 to 300 µM (9.375, 18.75, 37.5, 75, 150, and 300 µM) for 72 h. Additionally, a positive control drug (cisplatin) was used to test the compounds under the same experimental conditions to enable comparison with the cell lines. After 72 h of incubation, the medium was removed, and MTT solution (50 µL, 5 mg/mL) was added to wells and incubated for 2 h. The medium was then discarded, and formazan crystals were dissolved by adding 200 µL of DMSO wells. The plate was shaken for 30 min to ensure complete dissolution. Absorbance was measured at 590 nm using an Epoch 2 ELISA microplate reader. IC_50_ values for **3a**, **3b** and cisplatin were determined using GraphPad Prism (version 5). Selectivity indexes (SI) were calculated by dividing the IC_50_ values obtained from healthy cells (HEK-293T) by the IC_50_ values from cancer cell lines (A549, HepG2, and MCF7) for each compound.

### RT-qPCR

RT-qPCR experiments were conducted as previously reported [33]. Total RNA was extracted from cells using a PureLink™ RNA Mini Kit (Invitrogen, #12183018A) following the manufacturer’s instructions, and residual genomic DNA was removed by treatment with DNase I (New England Biolabs, #M0303S). Complementary DNA (cDNA) was synthesized using the iScript™ cDNA Synthesis Kit. Quantitative real-time PCR was conducted via SsoAdvanced™ Universal SYBR^®^ Green Supermix on a CFX Connect Real-Time PCR system following the manufacturer’s recommendations. Primer pairs targeting *BAX*, *BCL-2*, *BiD*, *BCL-xL*, and the reference gene *ACTB* were employed; primer sequences are available upon request. Relative gene expression levels were determined using the 2^−ΔΔCt^ (Livak) method [34], with normalization to *ACTB* expression in each sample. Amplification specificity was confirmed by melting curve analysis. All qRT-PCR experiments were conducted three times, including both technical and biological replicates. Results were expressed as fold change values and presented graphically as mean ± SD.

### DAPI staining

Cells were seeded onto coverslips in 12-well plates at a density of 1.25 × 10^5^ cells per well. After treatment with the indicated compounds for 72 h, cells were washed with ice-cold 1× PBS and fixed using 4% formaldehyde in PBS. Following fixation, nonspecific binding sites were blocked with BSA, and nuclei were stained with DAPI (4′,6-diamidino-2-phenylindole dihydrochloride). Nuclear morphology was subsequently examined using a fluorescence microscope (Olympus EX71). Staurosporine was provided from Cell Signaling Technologies (#9953) and used as a positive control of apoptotic induction. All procedures were performed in three independent biological replicates.

### β-galactosidase assay

Cellular senescence assays were conducted as previously explained [35]. Cells were seeded in 12-well plates and treated with the indicated compounds for 48 h. After treatment, the culture medium was removed, and cells were washed twice with ice-cold 1× DPBS before being fixed with 4% formaldehyde in PBS. Cells were subsequently incubated in X-gal staining solution at 37 °C for 48 h, washed twice with 1× DPBS, and refixed. Senescent cells exhibiting green–blue staining were counted to determine the senescence rate. All experiments were conducted in three biological replicates, and data are presented as fold-change values expressed as mean ± SD.

### Total oxidant status (TOS)

Total oxidant status (TOS) was determined spectrophotometrically using a commercial assay following the manufacturer’s protocol. The method is based on the oxidation of ferrous ions to ferric ions by various oxidant species in an acidic medium, with the resulting ferric ions quantified using xylenol orange. Following 48 h of treatment with the selected compounds at IC_50_ concentrations, oxidant status was determined.

### Total antioxidant status (TAS)

TAS was measured spectrophotometrically using a commercial assay (REL Assay Diagnostics^®^) according to the manufacturer’s instructions. Cells were treated with the selected compounds at their IC_50_ concentrations for 48 h, after which TAS was determined. The assay was calibrated using Trolox, and TAS values were expressed as micromoles of Trolox equivalents per liter (μmol Trolox eq/L).

### DNA/BSA studies

An initial solution of CT-DNA and BSA was prepared via dissolving an accurately weighed amount of DNA and BSA in Tris–HCl buffer (pH 7.2) to obtain a 1.5 mg/mL concentration (for DNA) and 3 mg/mL concentration (for BSA). The solution was stirred to ensure complete dissolution and stored at 4 °C until further utilization. Stock solutions of compounds **3a** and **3b** (1.0 × 10^−5^ M) were prepared separately in ethanol.

#### Fluorescence titrations

Considering the cytotoxic and biological activity of compounds **3a** and **3b**, their interactions with DNA and BSA were examined via fluorescence spectroscopy. All measurements were performed at 298 K during the gradual addition of the compounds. For the DNA-binding experiments, emission spectra were noted over the range of 510–700 nm with an *λ*_*ex*_ of 471 nm. The assay mixture consisted of 170 µL of CT-DNA solution, 30 µL of ethidium bromide, and 2000 µL of Tris–HCl buffer, giving a final volume of 2200 µL. For the BSA-binding studies, 50 µL of BSA solution was mixed with 2000 µL of buffer to obtain a final volume of 2050 µL, and the emission spectra were collected in the range of 300–500 nm upon *λ*_*ex*_ at 295 nm. Binding interactions were evaluated by stepwise titration of **3a** and **3b** over the concentration range of 0–1.40 µM (for **3a**+CT-DNA), 0–1.40 µM (for **3b**+CT-DNA), 0–1.16 (for **3a**+BSA), and 0–1.16 µM (for **3b**+BSA). After each addition, the solutions were allowed to equilibrate for 2 min before recording the spectra to ensure stabilization of the ligand+biomolecule interactions. For the synthesized compounds, relatively low concentration ranges were selected for the fluorescence measurements. At higher concentrations, the spectral changes became excessively pronounced, which could interfere with accurate analysis and reliable interpretation of the binding parameters. Therefore, lower concentrations were chosen to ensure controlled quenching behavior and linearity within the studied range. The fluorescence spectra were recorded using a SHIMADZU RF-5301PC spectrofluorophotometer.

### DFT and QTAIM details

The molecular structures of **3a** and **3b** were optimized using DFT with the B3LYP exchange-correlation functional. All calculations were performed in the gas phase using the Gaussian 09 W software package [36], and molecular structures were visualized using the GaussView 6.0 interface. A 6–311g (d) basis set was applied to all atoms. Following geometry optimization, MEP maps as well as HOMO–LUMO distributions were generated. The calculated orbital energies were subsequently used to determine a series of quantum chemical reactivity descriptors for the synthesized compounds. Furthermore, topology analyses such as RDG/RDG scatter, ELF, and LOL were performed using Multiwfn program.

### Docking studies

The potential biological property of the synthesized compounds, their anticancer activity, and DNA and BSA interactions were investigated through molecular docking simulations. The preferred binding orientations between the ligands and the biological target were predicted using AutoDock 4.2 [37] in conjunction with AutoDock Tools 1.5.6 [38]. The X-ray crystal structures of the selected cancer-related protein (PDB ID: 3ERT), DNA (PDB ID: 1Z3F), and BSA (PDB ID: 4JK4) were retrieved from the Protein Data Bank. Before starting the docking, the two proteins and DNA structures were prepared via deleting crystallographic water molecules and co-crystallized ligands. Kollman and Gasteiger charges and polar hydrogen atoms were then assigned to the proteins and DNA. A grid box was positioned to encompass the active site region with dimensions of 98 × 98 × 98 for 3ERT, 50 × 46 × 74 for 1Z3F, and 84 × 106 × 80 for 4JK4. Docking calculations were performed using the LGA. The resulting binding conformations were extracted from the output files and further examined by Discovery Studio Visualizer 4.1.

### Statistical analysis

Statistical data analysis was conducted using GraphPad Prism (Version 7; GraphPad Software, Boston, MA, USA). Results are expressed as fold change values with corresponding standard deviations. Statistical differences between groups were analyzed by Student’s t-test or one-way analysis of variance (ANOVA), followed by Tukey’s post hoc multiple comparisons test. A p-value of less than 0.05 (*p*<0.05) was considered statistically significant.

## Results and discussion

### Synthesis of benzimidazolium salts

**Scheme 1:**
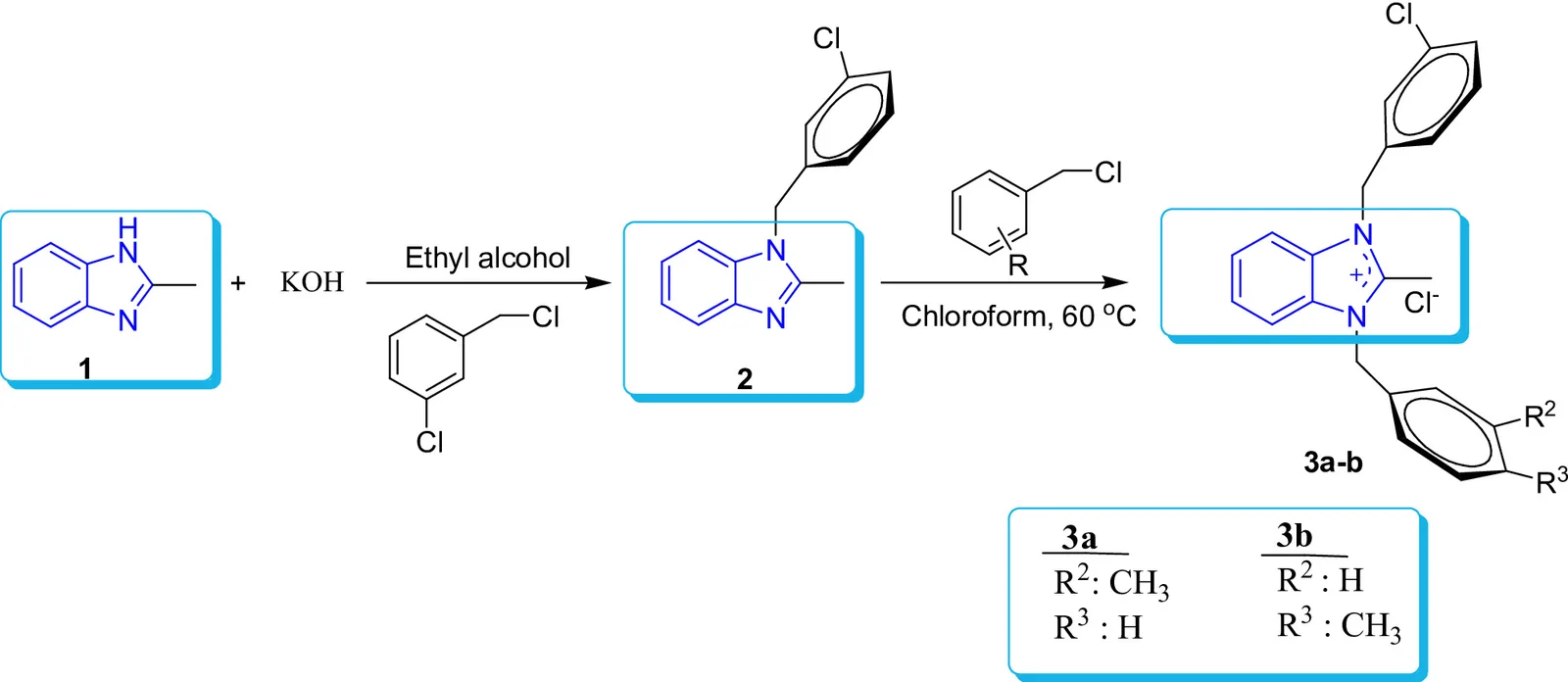
Synthesis diagram of compounds

In this investigation, two different benzimidazolium salts (**3a** and **3b**) were successfully synthesized (Scheme 1).

When the ^1^H NMR spectra were examined, it was determined that signals belonging to the methyl protons were observed around 2.28 and 3.01 ppm in both compounds, while the methylene (CH_2_) protons attached to the benzimidazole core gave signals in the range of approximately 5.75–5.87 ppm. Multiple signals belonging to aromatic protons were detected in the 7.15–7.97 ppm range. These data support that both compounds were synthesized as intended. In the ^13^C NMR spectra, the signals of the methyl carbons were detected in the range of approximately 11–21 ppm, while the signals of the methylene carbons were observed around 47–49 ppm. The signals belonging to the aromatic carbons and the NCN carbons in the benzimidazole ring were detected in the range of 113–153 ppm. The LC-MS/MS analysis performed for compound **3b** found the [M–Cl]⁺ ion at m/z = 361.05 g/mol, consistent with the calculated value of 361.90 g/mol, confirming the successful synthesis of the target molecule.

### Cytotoxic activity studies

The cytotoxic activities of **3a** and **3b** were evaluated in HEK-293T, A549, HepG2, and MCF-7 cell lines (Table 1). The results obtained were compared with cisplatin, which was used as a reference drug.

**Table 1:** IC_50_ values of the designed compounds against human cell lines

Compounds	IC_50_ (µM)			
	HEK-293T	A549	HepG2	MCF-7
**3a**	39.69	84.08	15.88	17.58
**3b**	17.75	51.16	10.83	11.55
Cisplatin	25.86	12.13	42.63	19.63

In the healthy HEK-293T cell line, the IC_50_ value of compound **3a** was determined to be 39.69 µM, while the IC_50_ value of compound **3b** was determined to be 17.75 µM. These results indicate that compound **3b** exhibits higher cytotoxicity on healthy cells compared to **3a**. The IC_50_ value of the reference drug cisplatin was found to be 25.86 µM. Accordingly, the toxicity of **3b** on healthy cells is higher than that of cisplatin, while that of **3a** is lower. This makes compound **3a** more advantageous in terms of selectivity.

**Fig. 2:**
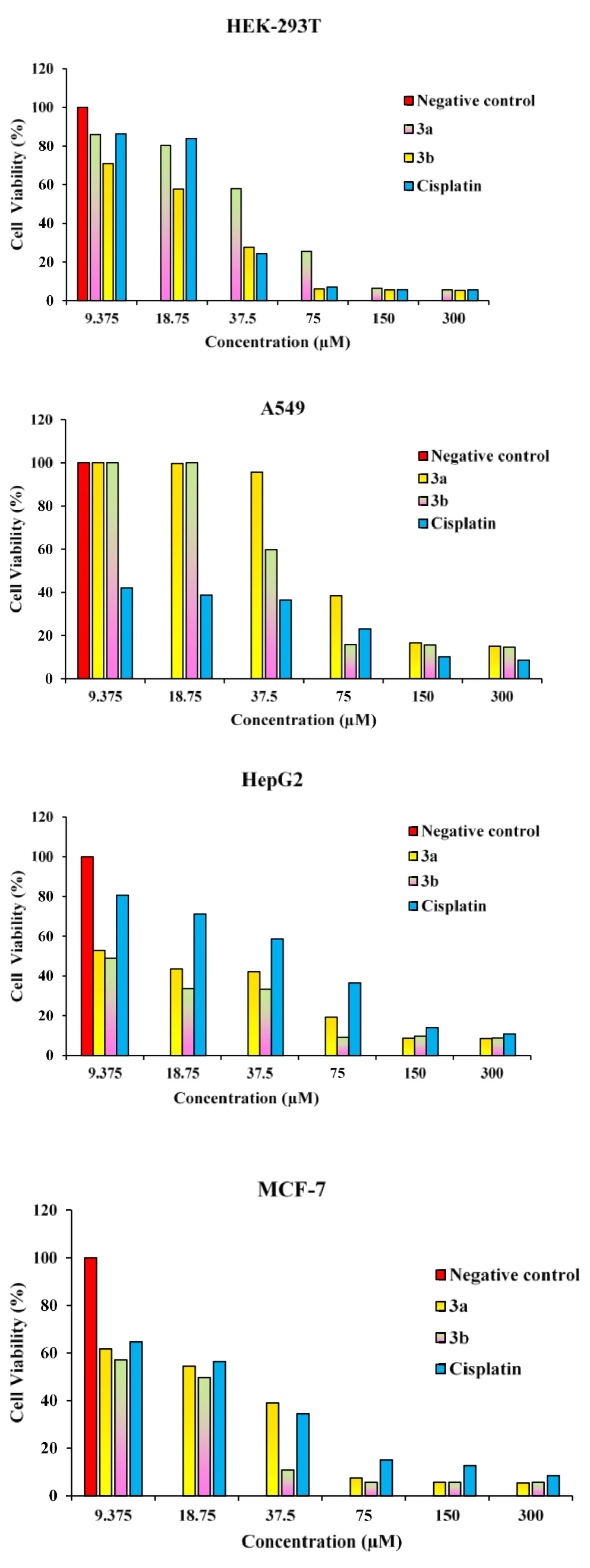
Cell viability ratio depends on concentrations of compounds

The IC_50_ values of **3a** and **3b** in the A549 cell line were calculated to be 84.08 µM and 51.16 µM, respectively. Compound **3a**, in particular, exhibited weak cytotoxic effects against A549 cells. These results indicate that the designed compounds have limited antiproliferative potential against the lung cancer cell line. Noteworthy results were obtained in the HepG2 liver cancer cell line. The IC_50_ value of compound **3a** was determined to be 15.88 µM, while that of compound **3b** was 10.83 µM. These values are significantly lower than the IC_50_ value of cisplatin in HepG2 cells (42.63 µM). Therefore, both compounds exhibited a stronger cytotoxic effect in the liver cancer cell line compared to the reference drug. Compound **3b**, in particular, stands out as the compound exhibiting the highest activity in this cell line. The IC_50_ values of **3a** and **3b** in the MCF-7 cell line were calculated as 17.58 µM and 11.55 µM, respectively. Based on these results, both compounds exhibited stronger antiproliferative effects than cisplatin. In particular, compound **3b** demonstrated the lowest IC_50_ value in the MCF-7 cell line and showed promising activity against breast cancer cells. The concentration-dependent changes in the compounds are shown in Fig. 2.

The SI is an important parameter used to evaluate a compound’s selective cytotoxic effect on cancer cells compared to normal cells. The SI value is calculated by dividing the IC_50_ value determined in normal cells by the IC_50_ value in cancer cells (Table 2). High SI values indicate that the compound is more selective against cancer cells and exhibits lower toxicity toward normal cells.

**Table 2:** Selectivity index values of tested compounds on cells

Compounds	Cells			
	HEK-293T	A549	HepG2	MCF-7
**3a**	1	0.47	2.50	2.26
**3b**	1	0.35	1.64	1.54
Cisplatin	1	2.13	0.61	1.32

An evaluation of the selectivity index results reveals that compounds **3a** and **3b** exhibit varying degrees of selective cytotoxic activity against different cancer cell lines. Compound **3a** exhibited higher selectivity, particularly in HepG2 and MCF-7 cells, with SI values of 2.50 and 2.26, respectively; this indicates that the compound is more effective against cancer cells compared to normal HEK-293T cells. In contrast, the SI value of 0.47 obtained in A549 cells indicates low selectivity. Compound **3b** was found to exhibit moderate selectivity in HepG2 and MCF-7 cells, with SI values of 1.64 and 1.54, respectively. However, the SI value of 0.35 in A549 cells indicates lower selectivity against normal cells in this cell line. When cisplatin, a standard drug, was examined, the highest selectivity index was observed in A549 cells as SI=2.13.

### Assessment of the apoptotic effects of the compounds

**Fig. 3:**
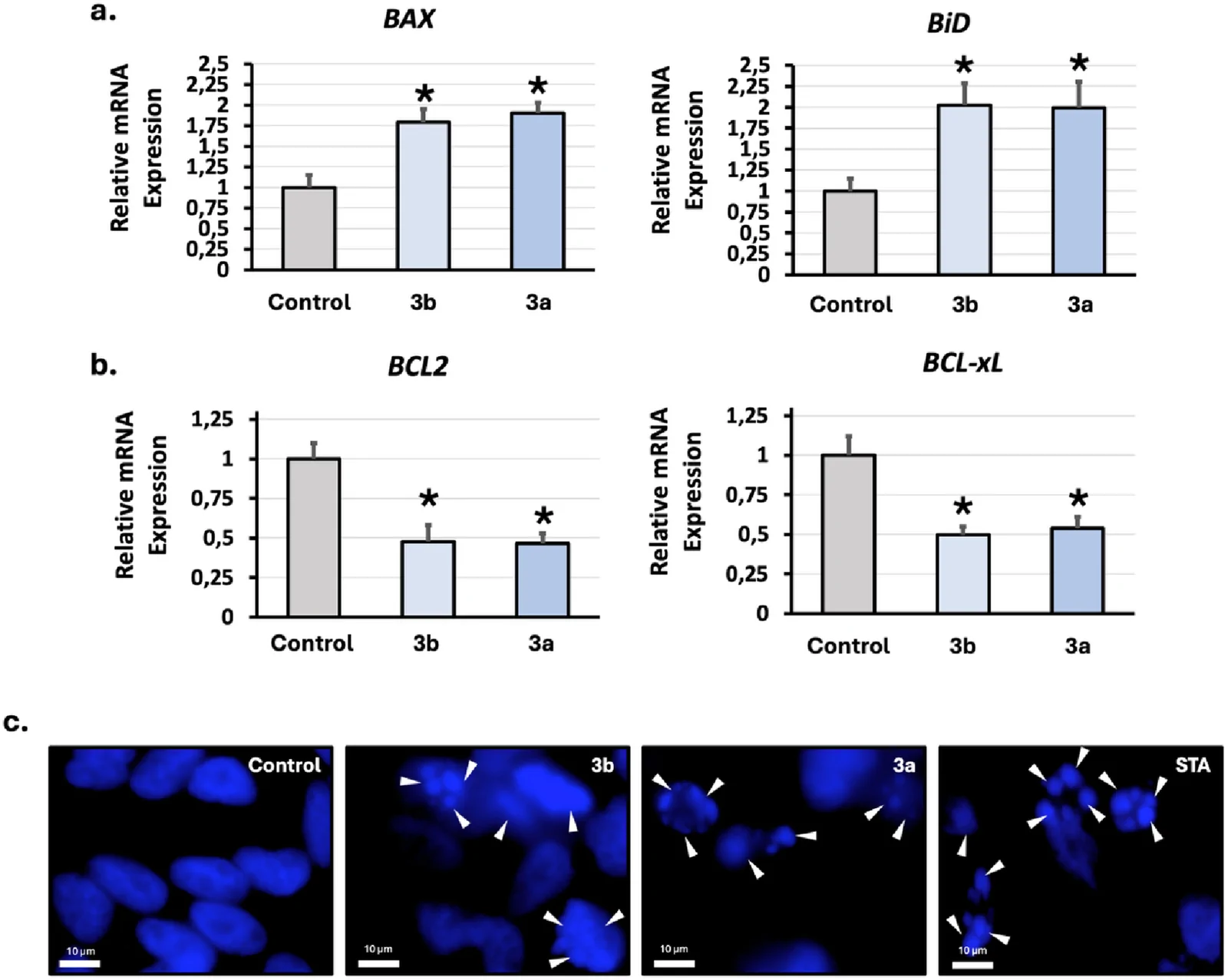
Apoptosis induction in MCF-7 cells treated with compounds at IC_50_ concentrations for 48 h. **a**, **b** RT-qPCR analysis of pro-apoptotic (*BAX*, *BiD*) and anti-apoptotic (*BCL2*, *BCL-xL*) gene expression relative to *ACTB*. Data are presented as fold change ± SD (*n* = 3); statistical significance was determined by two-tailed Student’s *t*-test (*p* < 0.05, *p* < 0.005). **c** Nuclear morphology was evaluated by DAPI staining and fluorescence microscopy; arrows indicate apoptotic bodies and nuclear fragmentation. 1 nM staurosporine (STA) was used as a positive control of apoptotic induction (*n* = 3; scale bar = 10 μm)

To elucidate the anticancer effect of compounds, mRNA expression levels of several apoptotic regulatory genes, including pro-apoptotic genes *BAX* and *BiD*, and anti-apoptotic *BCL2* and *BCL-xL* were analyzed in MCF-7 cells by RT-qPCR studies. Our data indicated that all tested compounds significantly up-regulated pro-apoptotic *BAX* and *BiD* mRNA levels compared to the control group. Antiapoptotic *BCL2* and *BCL-xL* were also significantly down-regulated in MCF-7 cells (Fig. 3a, b).

In studies, we aimed to test the apoptosis-inducing effects of compounds by examining changes in nuclear morphology through DAPI staining studies. Consistent with RT-qPCR results, we found that both compounds caused dissociation and condensation of the nuclear structures in MCF-7 cells (Fig. 3c). These findings suggested that **3a** and **3b** triggered the apoptotic induction in MCF-7 cells.

A significant portion of the developed natural or synthetic anticancer agents are known to induce apoptotic cell death in cells through various pathways. In this way, the growth, development, and multiplication of cancer cells can be prevented [39–41]. In mammalian cells, changes in the balance between pro-apoptotic and anti-apoptotic genes, such as *BAX* and *BiD*, *BCL2* and *BCL-xL*, are a decisive mechanism in regulating the apoptotic process, determining the ultimate fate of the cells [30, 42]. Herein, to understand the anticancer mechanism of compounds, their effect on apoptosis-related genes in MCF-7 cells was evaluated. The findings indicated that compounds importantly induced the expression levels of pro-apoptotic *BAX* and *BiD* and, conversely, decreased the antiapoptotic *BCL2* and *BCL-xL* (Fig. 3). These results suggest that the tested compounds exhibit anticancer effects on cancer cells through modulating apoptotic genes.

### Oxidative stress and senescence in MCF-7

**Fig. 4:**
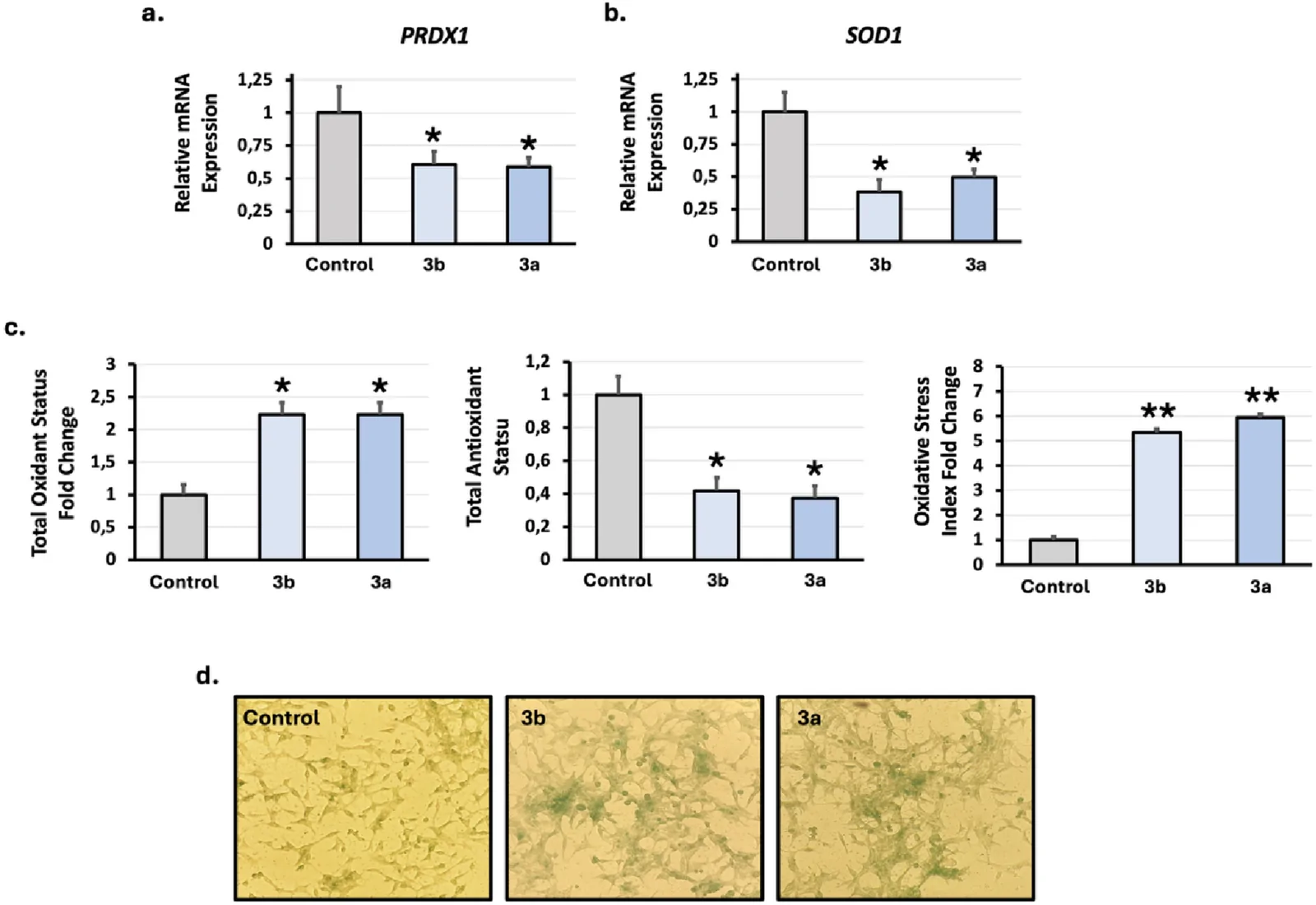
Effects of selected compounds on oxidative stress and cellular senescence in MCF-7 cells treated at IC_50_ concentrations for 48 h. **a**, **b** RT-qPCR analysis of antioxidant-related genes (*PRDX1* and *SOD1*) relative to *ACTB*; data are shown as fold change ± SD (*n* = 3), with statistical significance determined by two-tailed Student’s *t*-test (*p* < 0.05). **c** Oxidative stress parameters were measured spectrophotometrically, including TAS, TOS, and the oxidative stress index (OSI = TOS/TAS); data are presented as fold change ± SD (*n* = 3; *p* < 0.05, *p* < 0.001). **d** Cellular senescence was evaluated by X-gal staining, as described in the experimental section

We aimed to investigate the effects of compounds on cellular oxidative status in MCF-7 cells. Herewith, mRNA expression levels of antioxidant system-related PRDX1 and SOD1 were analyzed by RT-qPCR studies. Total antioxidant and total oxidant levels were also examined by TAS and TOS analysis, and the oxidative stress index was determined. We found that PRDX1 and SOD1 genes were significantly down-regulated by the compounds compared to the control group (Fig. 4a, b). Furthermore, treatment with the compounds led to a significant increase in the total oxidant status of MCF-7 cells, accompanied by a marked decrease in total antioxidant status relative to the control (Fig. 4c). When the cellular oxidative stress index was calculated, it was determined that the compounds significantly increased oxidative stress levels in MCF-7 cells (Fig. 4c).

Increased oxidative stress is known to be closely related to cellular aging [43]. Therefore, to determine the effect of compounds on cellular aging, we performed the senescence-associated β-galactosidase assay. Consistent with the previous oxidative stress data, senescence results revealed that compounds importantly induced cellular senescence in MCF-7 compared to the control group. It was observed that the compounds significantly increased green staining depending on the β-galactosidase activity in the cells (Fig. 4d). As a result, these findings indicate that compounds **3a** and **3b** enhanced oxidative stress, which in turn contributed to the induction of cellular senescence in MCF-7 cells.

Anticancer agents disrupt redox processes in numerous cancer cells, leading to increased oxidative conditions [44–46]. This, in turn, causes increased cellular stress and disrupted homeostatic mechanisms, driving cells towards programmed cell death [47]. In our studies aimed at understanding the anticancer mechanism of these substances, we tested their effects on cellular oxidative stress balance. Our results showed that both agents suppressed total antioxidant levels while increasing oxidative levels. As part of the investigation, we assessed mRNA expression of *PRDX1*, a member of the peroxiredoxin family that functions to detoxify hydrogen peroxide and alkyl hydroperoxides [48]. Moreover, we examined the mRNA levels of SOD1, one of the enzymes responsible for the elimination of superoxide [49]. Our findings supported the oxidant status experiments. Surprisingly, compounds significantly reduced the mRNA expression levels of both antioxidant enzymes (Fig. 4). These results suggest that the tested compounds reprogrammed enzyme levels associated with the antioxidant system in cancer cells, leading to increased oxidative stress and potentially stimulating programmed cell death mechanisms in these cells. It is also known that oxidative stress drives cellular aging processes in cells. Our findings indicate that both compounds stimulate cellular senescence, suggesting that the tested compounds exert effects on cellular senescence, either directly or through alternative mechanisms, as part of their anticancer mechanisms (Fig. 4). More in-depth characterization of the anticancer mechanisms of these compounds requires a comprehensive investigation of molecular signaling mechanisms using advanced experimental models.

When all findings are considered together, it is thought that the anticancer effect of the tested substances is due to disrupting intracellular redox balance, leading to increased oxidative stress; consequently, the increased reactive oxygen species cause DNA and protein damage, limiting the viability of cancer cells. Furthermore, our findings suggest that cellular senescence can be induced through cell cycle regulators due to oxidative stress induced in cells, and ultimately, anticancer effects emerge through the triggering of apoptosis. In this context, our findings mechanistically suggest that the anticancer effect of the tested substances on cells directs cell fate through cellular senescence and apoptosis, which are induced by increased oxidative stress.

### Macromolecules’ interactions

#### Fluorescence investigation of DNA binding

**Fig. 5:**
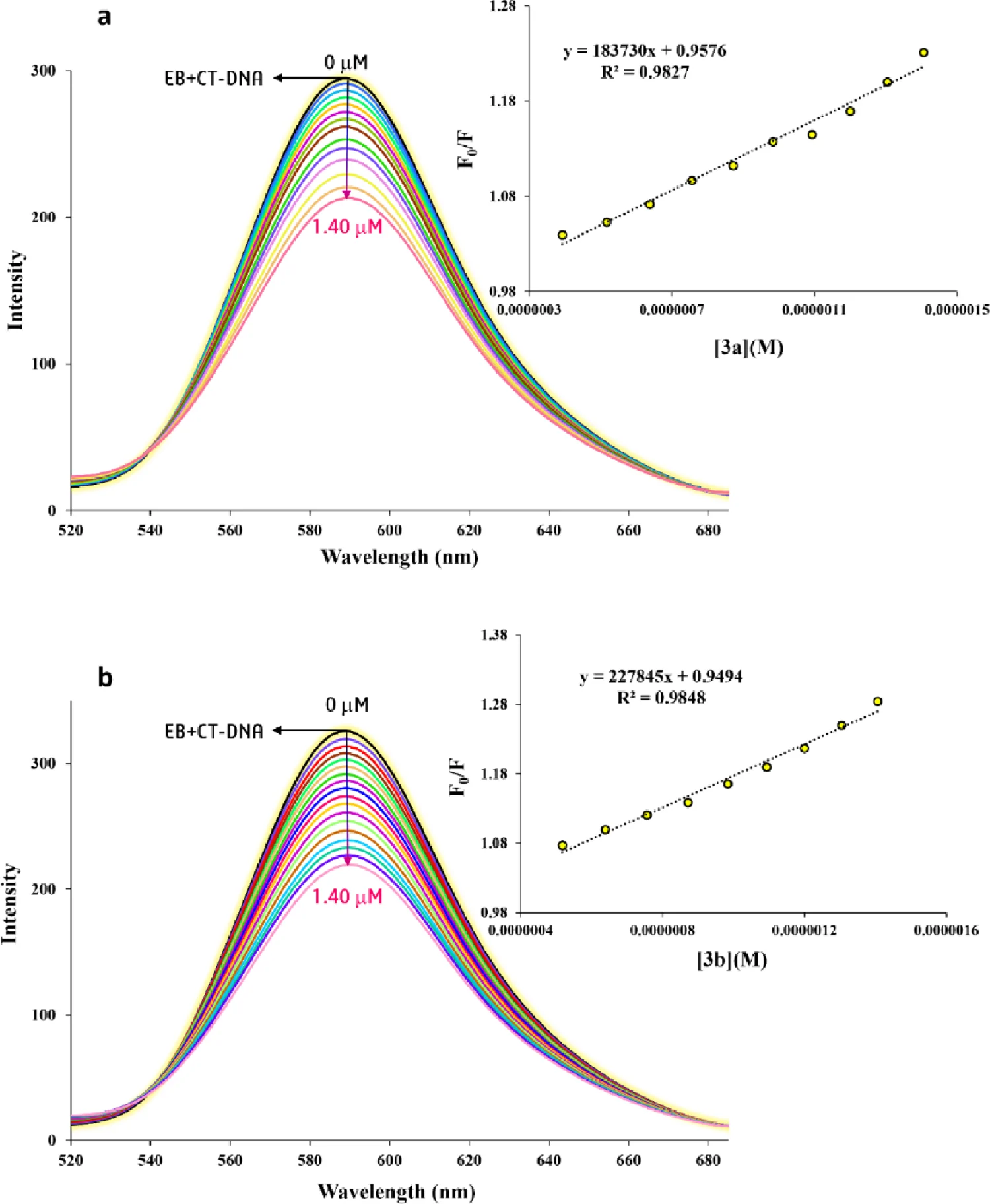
Fluorescence response of EB+CT-DNA without and with **3a** (**a**) or **3b** (**b**). Inset: Stern-Volmer plot at 298 K

Fluorescence titration was performed to investigate the interaction of **3a** and **3b** with CT-DNA. Small molecules can associate with DNA through either covalent or noncovalent interactions. Noncovalent binding generally occurs via three principal modes: intercalation between base pairs, groove binding, or electrostatic attraction to the phosphate backbone due to the negative charge [50]. Because native DNA exhibits negligible intrinsic fluorescence, EB was used as a fluorescent probe. EB strongly fluoresces upon intercalation into the DNA base pairs, producing a characteristic emission band around 600 nm. When a competing molecule interacts with DNA, it can displace or perturb the bound EB, resulting in a decrease in fluorescence intensity. As illustrated in Fig. 5, gradual addition of compounds **3a** and **3b** to the EB+CT-DNA system led to a progressive reduction in fluorescence emission intensity. In this context, quenching refers to the decrease in intensity of the fluorophore (EB+CT-DNA complex) upon addition of a quencher molecule. This reduction arises from changes in the microenvironment of the fluorophore and provides valuable information regarding the binding mechanism between the ligand and DNA [51]. The extent of fluorescence quenching depends on the ability of the compounds to displace intercalated EB from the hydrophobic DNA interior into the surrounding aqueous medium. Fluorescence quenching can occur through dynamic or static types, or through a combination of both processes [52]. These mechanisms can be distinguished by evaluating the quenching rate constant (*K*_*q*_) and comparing it with the maximum diffusion-controlled rate constant for dynamic quenching (approximately 2 × 10^10^ M^−1^ s^−1^). Values significantly exceeding this limit generally indicate a static quenching mechanism.

To clarify the quenching mechanism, the titration data obtained for the EB–CT-DNA and BSA systems in the presence of compounds **3a** and **3b** were analyzed utilizing the Stern–Volmer Eq. (1):

1 $$\:\frac{{F}_{o}}{F}=1+{K}_{SV}\left[3\boldsymbol{a}\:or\:3\boldsymbol{b}\right]$$

Here, *F*_*0*_ and *F* represent the fluorescence intensities of the macromolecule in the absence and presence of the quencher, respectively, and *K*_*SV*_ is the Stern–Volmer quenching constant; and *[****3a*** *or* ***3b****]* corresponds to the concentration of **3a** or **3b**. The values of *K*_SV_ are determined from the slope of the linear plots of *F*_0_/*F vs.* concentration of **3a** or **3b**. The apparent bimolecular quenching rate constant (*k*_q_) was predicted using *k*_q_= *K*_SV_/τ_0_, where τ_0_ is the average fluorescence lifetime of EB (1.0 × 10^−8^ s) [53]. The Stern–Volmer plots exhibit good linearity (Fig. 5, insets), yielding *K*_SV_ values of 1.83 × 10^5^ M^−1^ for **3a** and 2.28 × 10^5^ M^−1^ for **3b** in their interaction with CT-DNA. The corresponding *k*_q_ values are calculated as 1.83 × 10^13^ and 2.28 × 10^13^ M^−1^ s^−1^ for **3a** and **3b**, respectively. Since these *k*_q_ values are several orders of magnitude higher than 2 × 10^10^ M^−1^ s^−1^, the observed fluorescence quenching is attributed to a static mechanism, indicating complex formation between the compounds and the DNA rather than purely collisional interactions.

To further investigate the binding characteristics of **3a** and **3b** with CT-DNA, the binding constant (*K*_*b*_) and the number of binding sites (*n*) were determined using the double-logarithmic Eq. (2):

2 $$\:\mathrm{log}\frac{{F}_{0}-F}{F}=n\mathrm{log}\left[3\boldsymbol{a}\:or\:3\boldsymbol{b}\right]+\mathrm{log}{K}_{b}$$

**Fig. 6:**
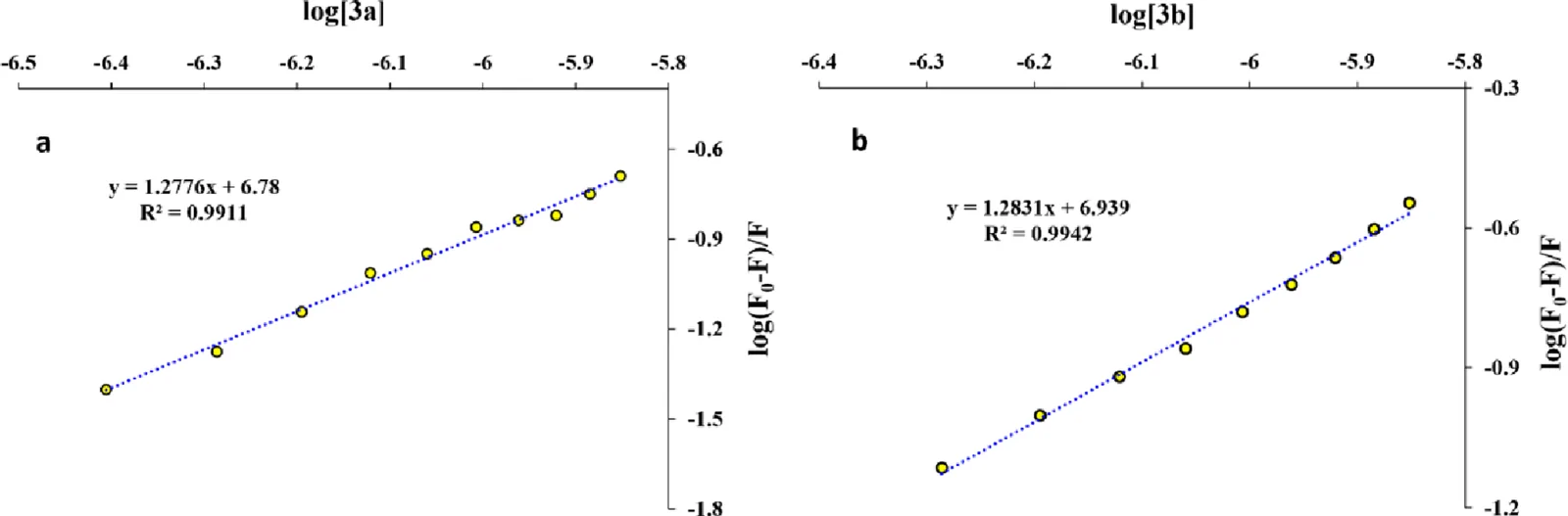
Diagram of *log(F*_*0*_*-F/F)* versus *log[****3a*** *or* ***3b****]* for **3a** (**a**) and **3b** (**b**) during the binding with CT-DNA at 298 K

From the linear plot of *log[(F*_*0*_*− F)/F]* versus *log[****3a****or****3b****]* (Fig. 6), the slope corresponds to the *n*, while the intercept gives the *K*_b_. The *n* values for **3a** and **3b** are 1.27 and 1.28, respectively, which are close to unity. This finding indicates an approximate 1:1 binding stoichiometry between the compounds and CT-DNA. The binding constants are determined to be 6.02 × 10^6^ M^-1^ for **3a** and 8.69 ×  10^6^ M^-1^ for **3b**, demonstrating strong affinity toward DNA. Notably, these *K*_b_ values are comparable to those reported for classical intercalators such as ethidium bromide (≈10^6^ M^-1^) [54], suggesting that both compounds may interact with DNA predominantly through an intercalative binding mode. The slightly higher binding constant observed for **3b** further supports its stronger interaction with DNA. The ΔG° for the binding process was calculated using the thermodynamic relation (3):

3 $$\:{\varDelta\:G}^{0}=-RT{LnK}_{b}$$

where *R* = 1.987 cal/molK and *T* = 298 K. The calculated ΔG° values are −9.24 kcal/mol for **3a** and −9.46 kcal/mol for **3b**, indicating that the binding processes are spontaneous under physiological conditions.

#### 3.5.2. Fluorescence investigation of BSA binding

**Fig. 7:**
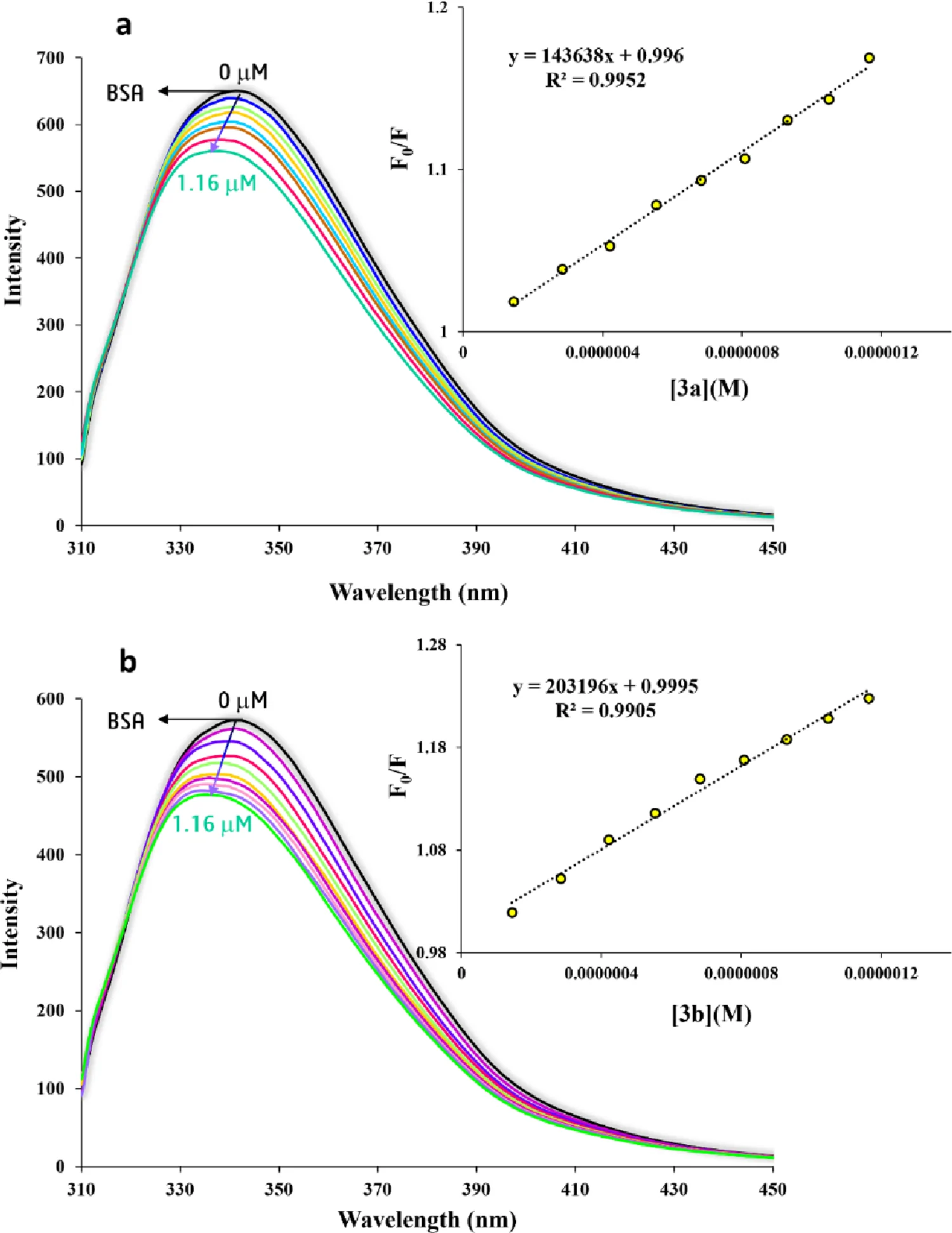
Fluorescence response of BSA without and with **3a** (**a**) or **3b** (**b**). Inset: Stern-Volmer plot at 298 K

The interaction of **3a** and **3b** with BSA was evaluated using fluorescence spectroscopy. Serum albumin was selected because it is the most abundant transport protein in blood plasma and plays a crucial role in drug distribution, bioavailability, and pharmacokinetics [55]. Understanding the binding behavior of small molecules toward BSA provides insight into their transport properties and potential systemic effects. Upon gradual addition of **3a** and **3b**, a progressive decrease in the intrinsic fluorescence intensity of BSA was detected (Fig. 7), indicating quenching of the tryptophan residues within the protein structure. The Stern–Volmer plots (Fig. 7, insets) showed good linearity, and the *K*_SV_ are calculated to be 1.43 × 10^5^ M^−1^ for **3a** and 2.03 × 10^5^ M^−1^ for **3b**. The corresponding *k*_q_ values are 1.43 × 10^13^ and 2.03 × 10^13^ M^−1^ s^−1^ for **3a** and **3b**, respectively. Since these values greatly exceed the 2 × 10^10^ M^−1^ s^−1^, the quenching mechanism is attributed to a static process, implying the formation of stable ground-state complexes between the compounds and BSA rather than purely dynamic (collisional) quenching. In addition, a slight blue shift in the emission maximum was detected after the addition of the compounds, suggesting changes in the microenvironment surrounding the tryptophan residues and a possible increase in hydrophobic character around the fluorophore.

**Fig. 8:**
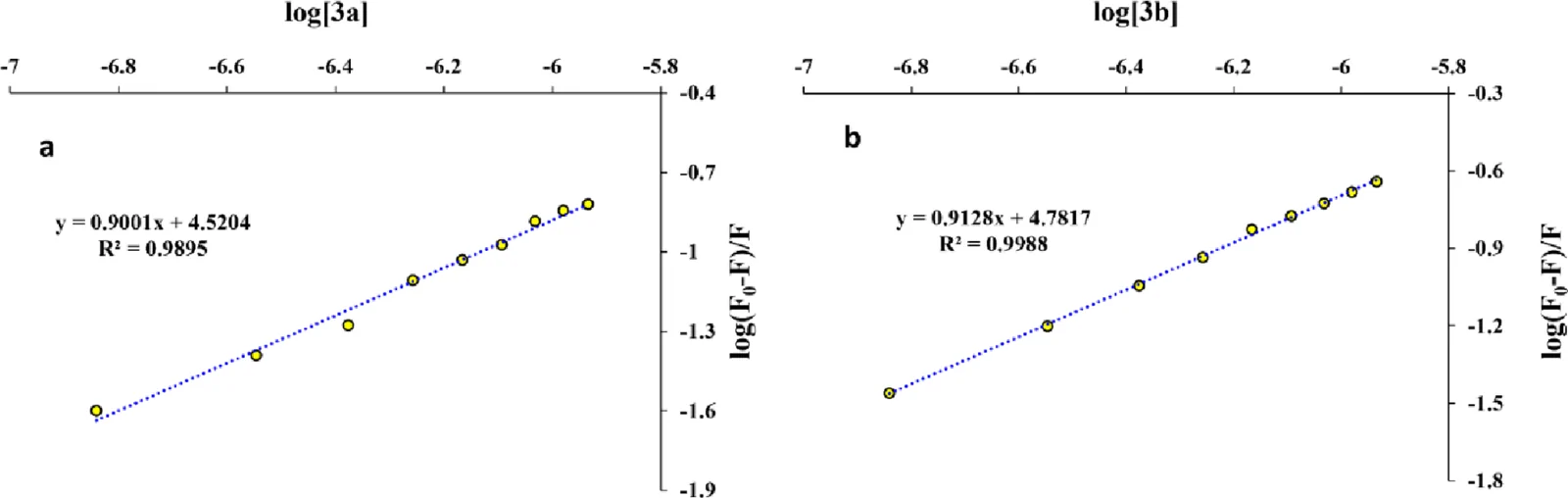
Diagram of *log(F*_*0*_*-F/F)* versus *log[****3a*** *or* ***3b****]* for **3a** (**a**) and **3b** (**b**) during the interaction with BSA at 298 K

Using the double-logarithmic equation (Eq. 2), *n* and *K*_b_ are determined (Fig. 8). The *n* values are calculated as 0.90 for **3a** and 0.91 for **3b**, indicating an approximate 1:1 binding stoichiometry between each compound and BSA. The interaction constants are found to be 3.31 × 10^4^ M^-1^ for **3a** and 6.05 × 10^4^ M^-1^ for **3b**, demonstrating moderate affinity toward serum albumin. Comparison of the DNA and BSA binding data clearly shows that both compounds exhibit significantly stronger affinity toward DNA than toward BSA. This preferential interaction with DNA may contribute to their anticancer activity, while the relatively moderate binding to serum albumin suggests limited nonspecific protein sequestration, which could be beneficial in reducing potential side effects.

The calculated ΔG° values are −6.16 kcal/mol for **3a** and −6.52 kcal/mol for **3b**, confirming that the binding processes are spontaneous under physiological conditions. The more negative ΔG° value for **3b** is consistent with its stronger binding affinity observed across both DNA and BSA systems.

### DFT details

#### Optimized geometry

**Fig. 9:**
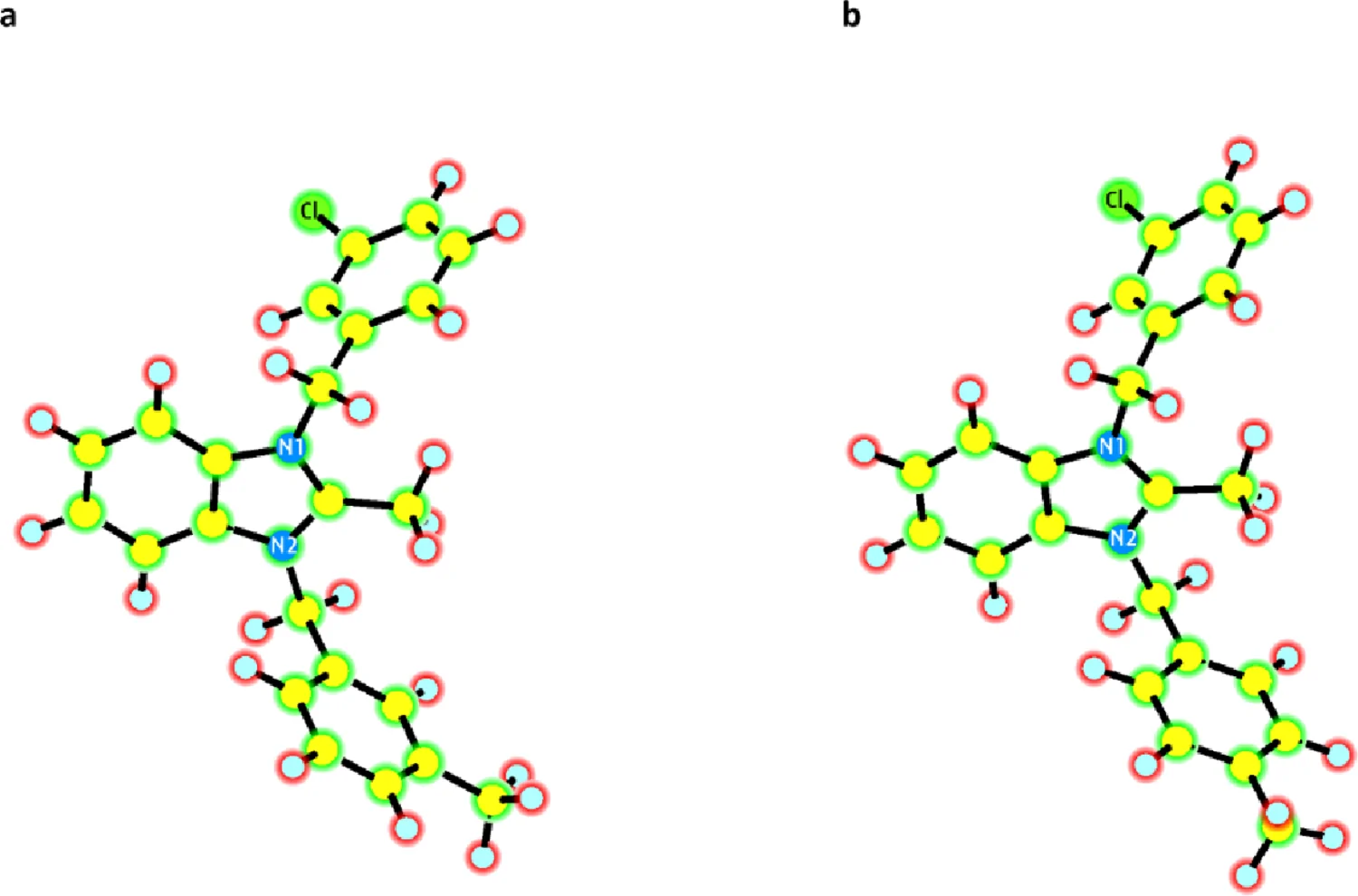
Suggested geometry of **3a** (**a**) and **3b** (**b**) by DFT

The optimized geometries of **3a** and **3b**, obtained at the B3LYP/6–311g (d) level of theory, showed no imaginary vibrational frequencies, confirming that both geometries correspond to true minima. The optimized molecular structures are presented in Fig. 9. The calculated total electronic energies are −1459.49 Hartree for **3a** and −1459.45 Hartree for **3b**. The dipole moments are determined to be 3.32 and 3.24 Debye for **3a** and **3b**, respectively. These moderate dipole moment values indicate a certain degree of lipophilic character, which may favor interactions with phospholipid bilayer membranes in biological environments and finally contribute to potential biological activity [56].

#### MEP surface

**Fig. 10:**
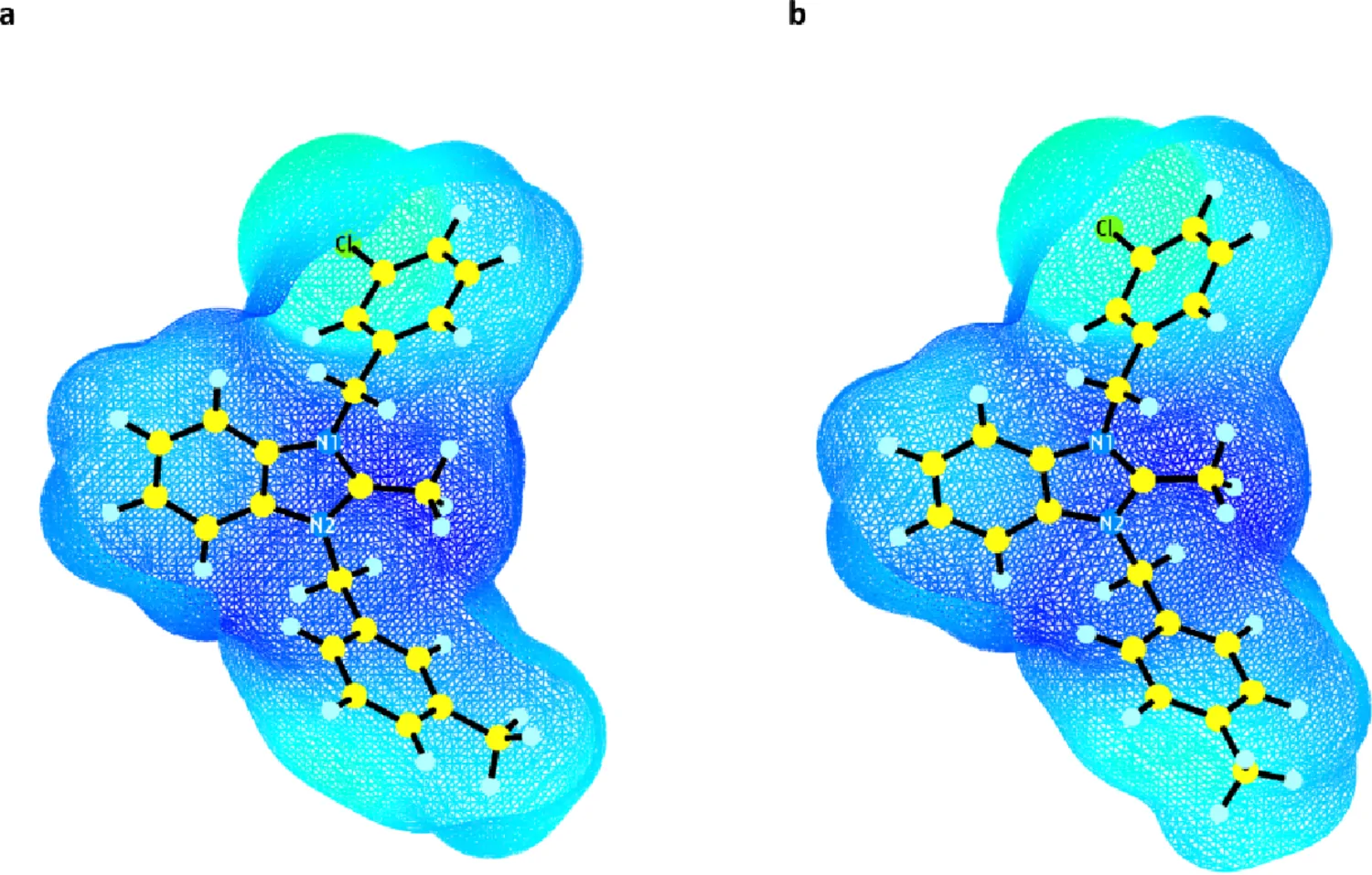
MEP diagram of **3a** (**a**) and **3b** (**b**)

The MEP surface, which provides a three-dimensional representation of the charge distribution within a molecule, offers valuable information about reactive regions and possible interaction sites. MEP analysis is useful for predicting hydrogen-bonding behavior as well as identifying preferred locations for nucleophilic and electrophilic attacks [57]. The electrostatic potential is visualized using a color scale ranging from red to blue: red corresponds to electron-rich areas with negative potential, blue indicates electron-deficient areas with positive potential, and white represents nearly neutral areas. Consequently, red regions are favorable sites for electrophilic attack, whereas blue regions are prone to nucleophilic attack [58]. The calculated MEP maps of compounds **3a** and **3b** are shown in Fig. 10. In both molecules, the positive electrostatic potential (blue regions) is broadly distributed across the molecular surface. This distribution arises from the absence of strongly electronegative heteroatoms such as oxygen or nitrogen exposed on the surface. As a result, the electron-deficient character dominates, suggesting that these molecules are more susceptible to nucleophilic attack.

#### Molecular orbitals and energy gap analysis

**Fig. 11:**
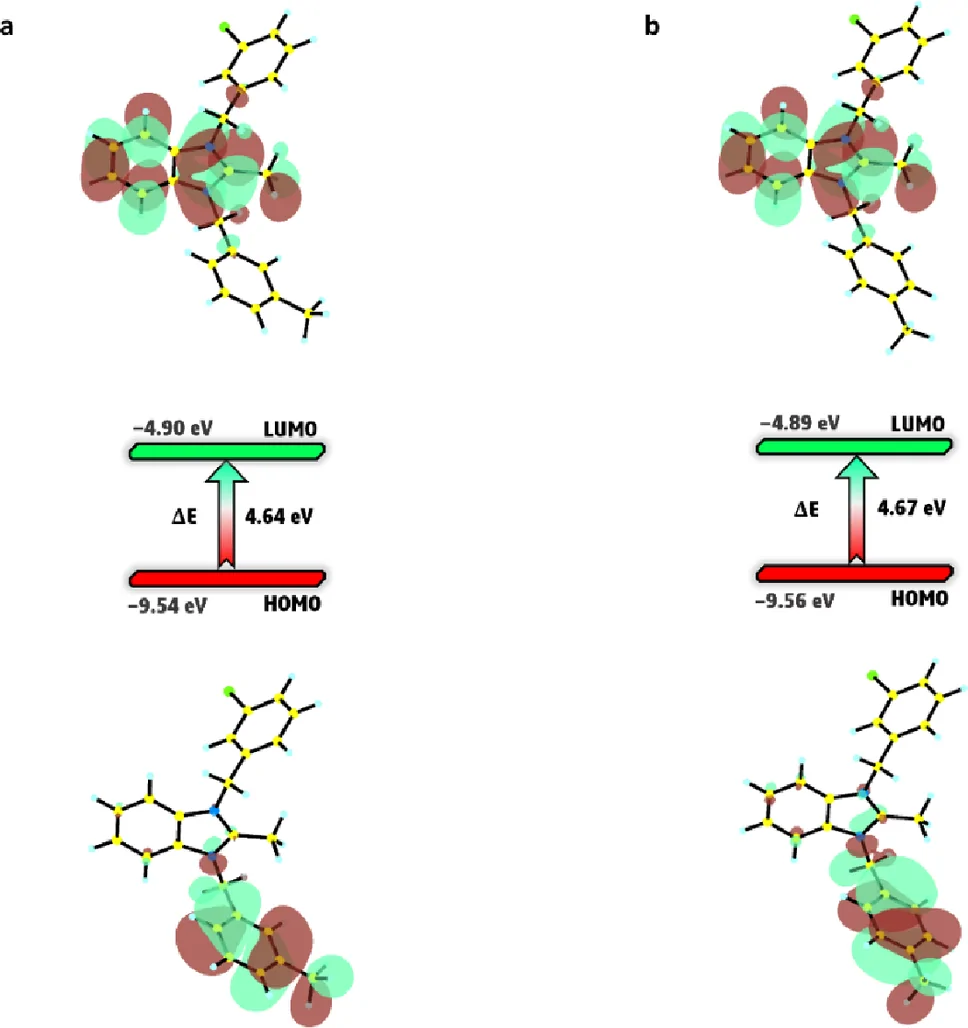
HOMO and LUMO distributions **3a** (**a**) and **3b** (**b**)

Fig. 11 shows the frontier molecular orbital (HOMO and LUMO) surfaces of **3a** and **3b**. These orbitals play a key role in governing molecular stability and chemical reactivity [59], where the HOMO acts as an electron donor and the LUMO as an electron acceptor. The energy gap of HOMO-LUMO (ΔE) is defined as follows:

4 $$\:\varDelta\:E={E}_{LUMO}-{E}_{HOMO}$$

This parameter serves as an important indicator of molecular reactivity and stability. A smaller energy gap facilitates electron transfer from the HOMO to the LUMO, generally corresponding to higher chemical reactivity and lower kinetic stability. The calculated energy gaps for **3a** and **3b** are 4.64 and 4.67 eV, respectively. These values fall within the typical range reported for biologically active molecules [57]. Moreover, compound **3a** exhibits slightly higher reactivity than **3b**, consistent with its marginally smaller energy gap. The spatial distributions of the HOMO and LUMO electron densities are largely similar for both compounds.

#### Quantum reactivity

Based on the calculated frontier orbital energies, several global reactivity descriptors were determined, including absolute electronegativity (χ), global hardness (η), chemical potential (μ), electrophilicity index (ω), global softness (σ), and the maximum electronic charge transfer (ΔN_max_). These parameters were evaluated using the following Eqs. (5–10):

5 $$\upmu=-{\upchi\:}$$

6 $$\:{\upchi\:}=\frac{-\left({E}_{HOMO}+\:{E}_{LUMO}\right)}{2}$$

7 $$\:{\upeta\:}=\frac{{E}_{LUMO}-{E}_{HOMO}}{2}$$

8 $$\:\sigma\:=\frac{1}{{\upeta\:}}$$

9 $$\:\omega\:=\frac{{\upmu}^{2}}{2{\upeta\:}}$$

10 $$\:{\varDelta\:N}_{max}=\frac{-\upmu}{{\upeta\:}}$$

The calculated quantum chemical descriptors for **3a** and **3b** are listed in Table 3. The electrophilicity index (ω) reflects the tendency of a compound to accept electrons from its surroundings. The relatively high ω values obtained for both compounds suggest a strong ability to interact with biological macromolecules through multiple non-covalent interactions. In addition, the negative chemical potential (μ) values indicate the stability of the investigated molecules. The parameter ΔN_max_ represents the maximum amount of electronic charge that a system can accept. The calculated values of 3.11 for **3a** and 3.10 for **3b** indicate a favorable capacity for charge transfer, which may facilitate intermolecular interactions with biomolecular targets.

**Table 3:** Quantum reactivity parameters of **3a** and **3b**

Parameter	3a	3b
E_HOMO_	−9.54	−9.56
E_LUMO_	−4.90	−4.89
ΔE_(LUMO-HOMO)_	4.64	4.67
χ	7.22	7.22
η	2.32	2.33
σ	0.43	0.43
µ	−7.22	−7.22
ω	11.23	11.18
ΔN_max_	3.11	3.10

#### RDG/RDG scatter analysis

**Fig. 12:**
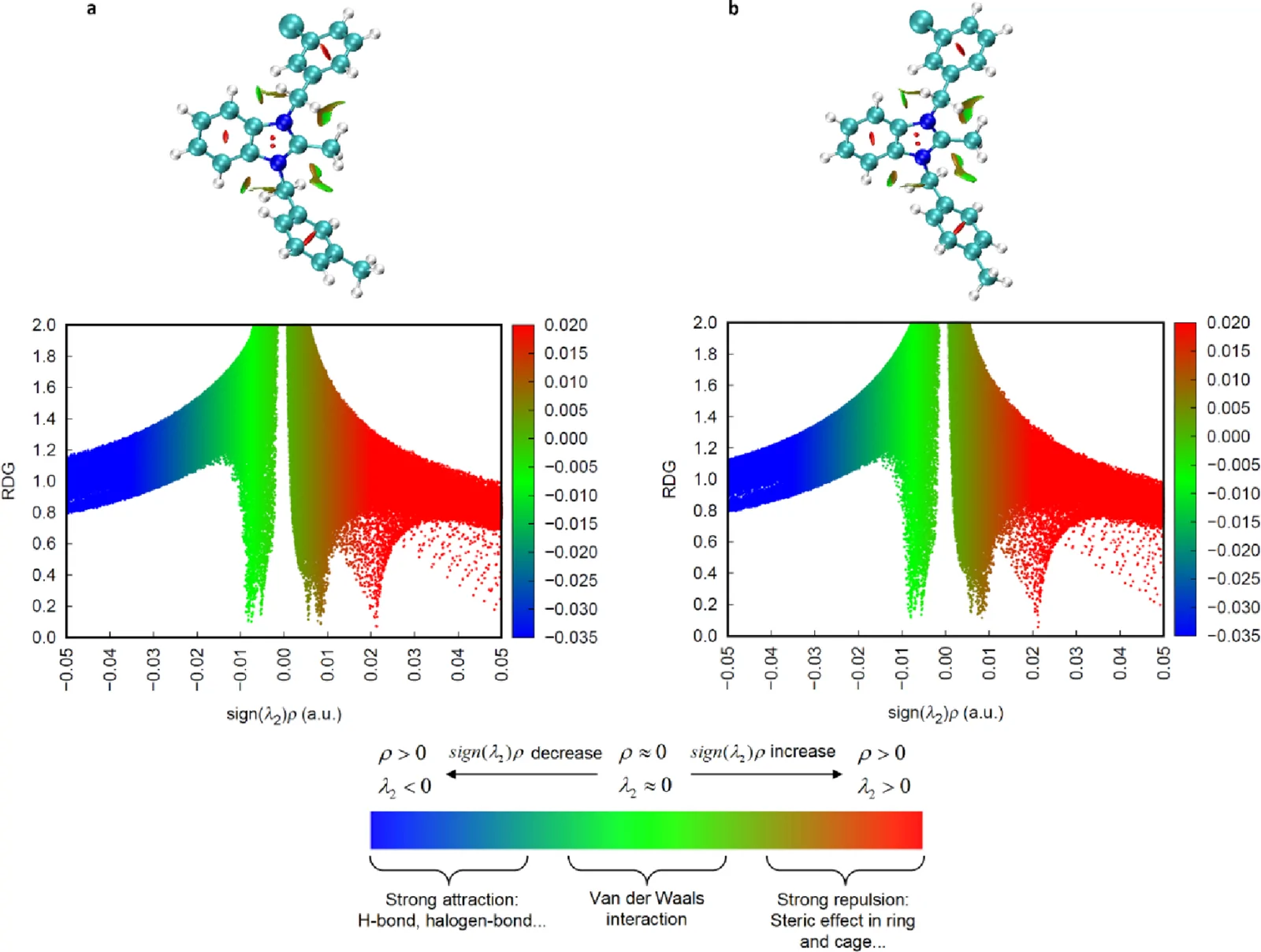
RDG/RDG scatter plot of **3a** (**a**) and **3b** (**b**)

To characterize the non-covalent interactions in the molecules, reduced density gradient (RDG) analysis based on electron density was performed. The nature of the interactions is interpreted through the sign of the second Hessian eigenvalue (*λ*_*2*_): negative *λ*_*2*_ values correspond to attractive interactions, positive *λ*_*2*_ values indicate steric repulsion, and values close to zero are associated with weak van der Waals interactions. A detailed examination was carried out by visualizing the RDG isosurfaces and analyzing the RDG scatter plots generated using the Multiwfn program for compounds **3a** and **3b** (Fig. 12). In the scatter plots, green regions represent weak non-covalent interactions, mainly dispersion-type contacts occurring at intermediate electron density. Both compounds exhibit pronounced green spikes, suggesting the presence of weak interactions such as C–H···C contacts, which may contribute to stabilization and could be relevant to their biological behavior [60]. Red regions observed on the RDG isosurfaces (approximately 0.015–0.05 a.u.) correspond to steric repulsion. These areas are mainly localized within the aromatic ring frameworks of the molecules, indicating steric congestion between adjacent aromatic carbon atoms.

#### ELF/LOL analysis

**Fig. 13:**
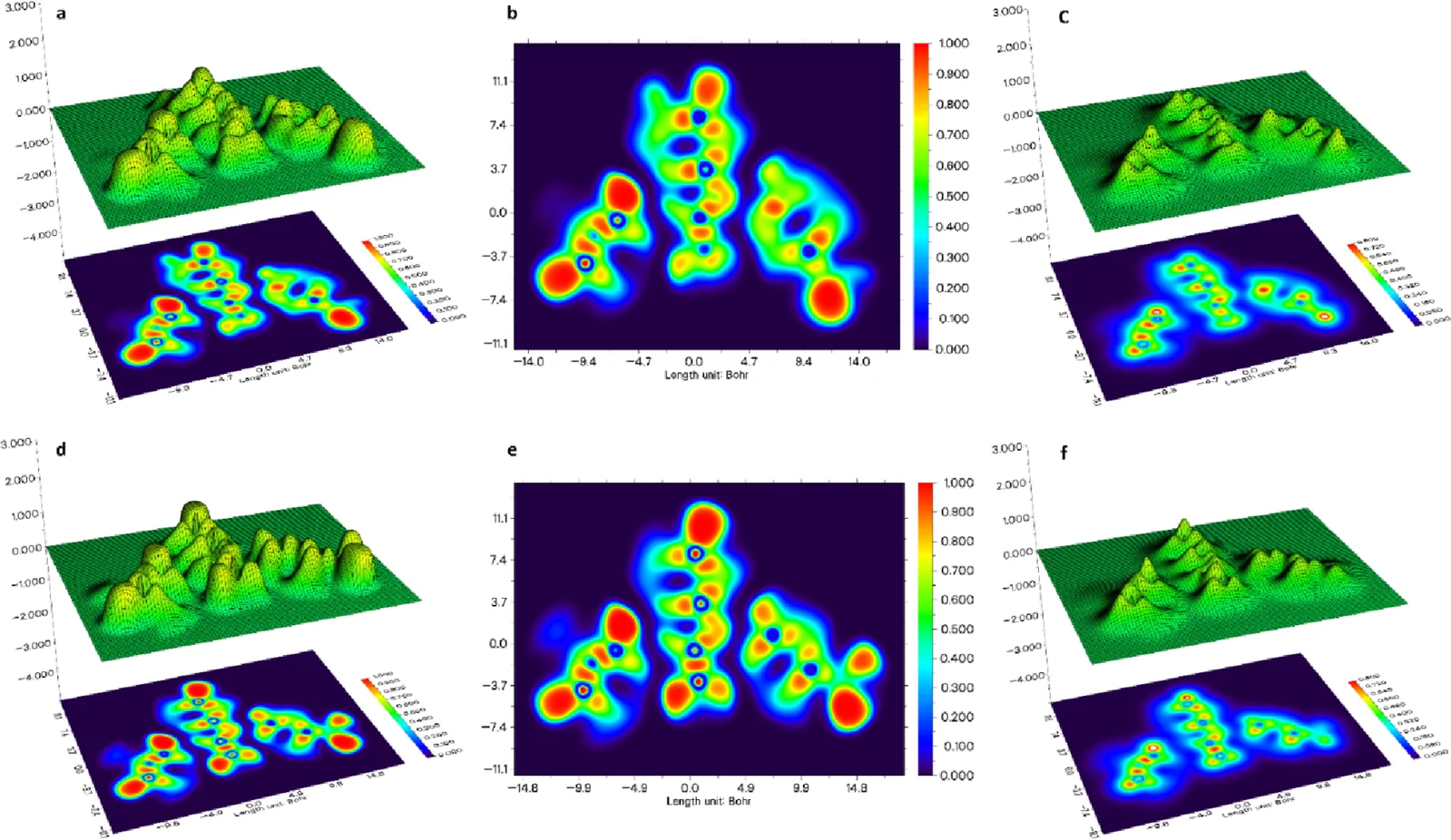
ELF (**a**, **b** for **3a** and d-e for **3b**) and LOL (c for **3a** and f for **3b**) plots

The electron localization function (ELF) describes the probability of finding two electrons with the same spin in close proximity within a given spatial region and is therefore useful for identifying bonding patterns and lone-pair regions. The ELF maps for compounds **3a** and **3b** are shown in Fig. 13 (a–b for **3a** and d–e for **3b**). The electron localization is visualized using a color scale ranging from red (high ELF values, 0.8–1.0) to blue (low ELF values). High ELF values (red regions) are mainly located along covalent bonds and in regions associated with localized electron pairs, reflecting strong electron localization within bonding domains. In contrast, blue regions indicate electron-delocalized areas, typically observed over π-conjugated frameworks and around atoms participating in extended electronic distribution, such as carbon, nitrogen, and chlorine atoms. The distribution pattern suggests that electrons are predominantly localized in σ-bonding regions, while π-systems exhibit partial delocalization across the molecular framework. This behavior is consistent with conjugated organic structures and provides insight into the electronic structure governing molecular stability and reactivity [60].

Complementary to the ELF analysis, the localized orbital locator (LOL) provides further insight into the degree of orbital localization and delocalization within the molecular framework. The LOL maps for compounds **3a** and **3b** are presented in Fig. 13 (c for **3a** and f for **3b**). The contour scale ranges from 0.00 to 0.80, where warm colors (red) indicate highly localized orbitals and cool colors (blue) represent regions of electron delocalization. In both molecules, red regions are mainly concentrated along σ-bonds, particularly around C–H bonding domains, reflecting localized bonding electron density. In contrast, carbon, nitrogen, and chlorine atoms within the conjugated framework appear predominantly in blue–green shades, indicating a more delocalized electronic structure associated with π-conjugation and electron distribution across the molecular skeleton. This distribution confirms the presence of localized σ-bonding regions together with extended electron delocalization over the aromatic/conjugated framework. The LOL results therefore support the electronic structure features inferred from the ELF analysis and provide additional evidence for the balance between localization and conjugation that governs molecular stability and reactivity.

### Docking studies

#### Anticancer activity

**Fig. 14:**
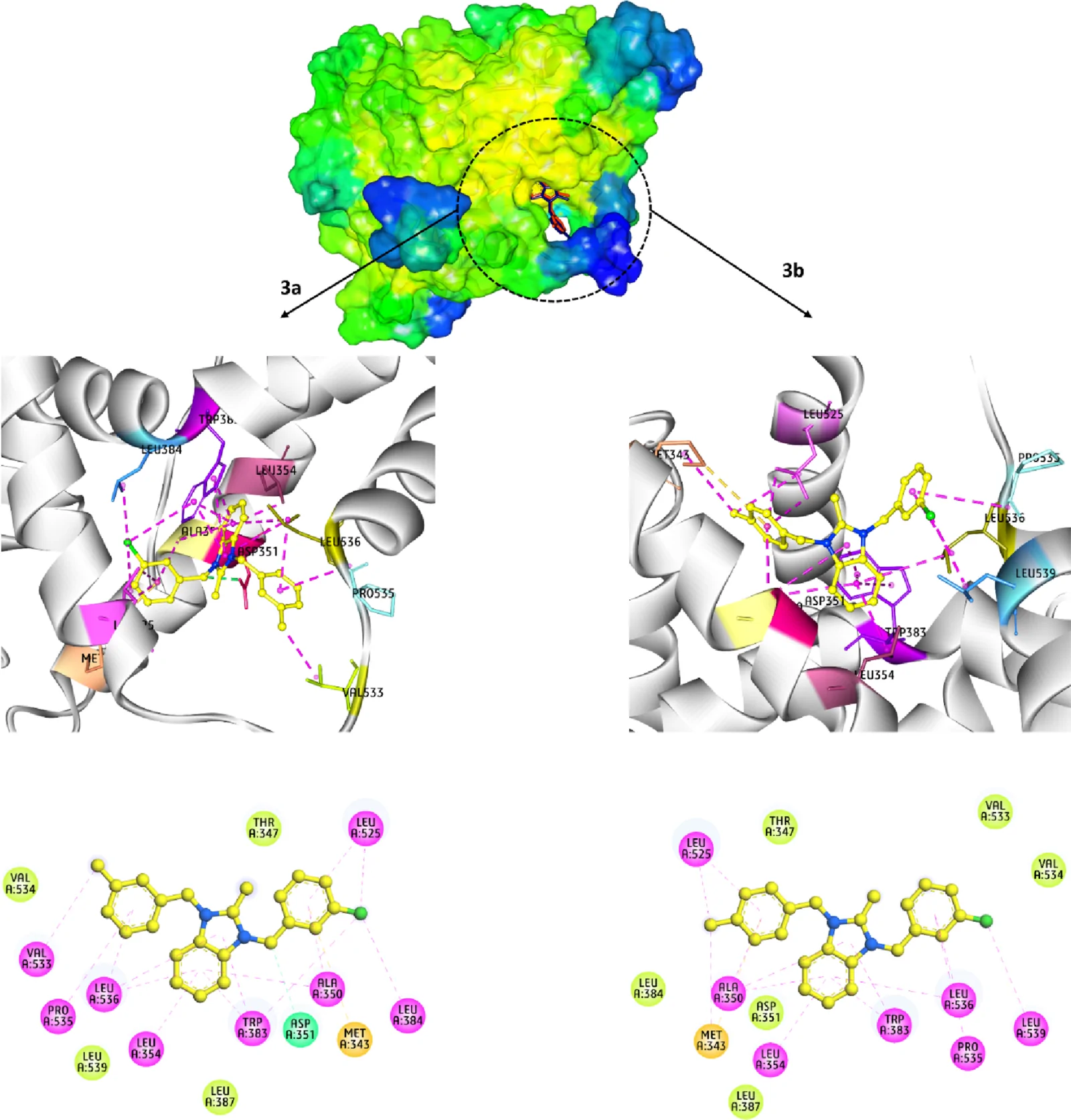
Docking finding of **3a** and **3b** in the binding with 3ERT

Considering the promising anticancer activity observed for compounds **3a** and **3b**, molecular docking simulations were performed to investigate their binding modes and interaction profiles with the target protein (PDB ID: 3ERT). The docking results reveal binding energies of −6.93 and −7.10 kcal/mol for **3a** and **3b**, respectively. These values indicate favorable binding within the active site of the protein. Compound **3b** exhibited a slightly stronger binding affinity than **3a**, which is consistent with the experimentally observed IC_50_ values obtained from the MTT assay. Figure 14 illustrates the predicted binding conformations of the ligands inside the binding pocket. Compound **3a** forms a hydrogen-bond interaction with ASP351 and establishes multiple hydrophobic contacts with VAL533, PRO535, LEU536, LEU354, TRP383, ALA350, LEU384, and LEU525. In addition, electrostatic interaction with MET343 and van der Waals contacts involving VAL534, LEU539, THR347, and LEU387 contribute to stabilization of the complex. Similarly, compound **3b** interacts through hydrophobic contacts with LEU525, ALA350, LEU354, MET343, TRP383, LEU536, PRO535, and LEU539, along with electrostatic interaction with MET343 and van der Waals interactions involving LEU384, ASP351, LEU387, THR347, VAL533, and VAL534. Hydrophobic contacts together with van der Waals interactions appear to play dominant roles in stabilizing the ligand–protein complexes, while hydrogen bonding provides additional directional stabilization. These results support the experimentally observed biological activity.

#### DNA interaction

**Fig. 15:**
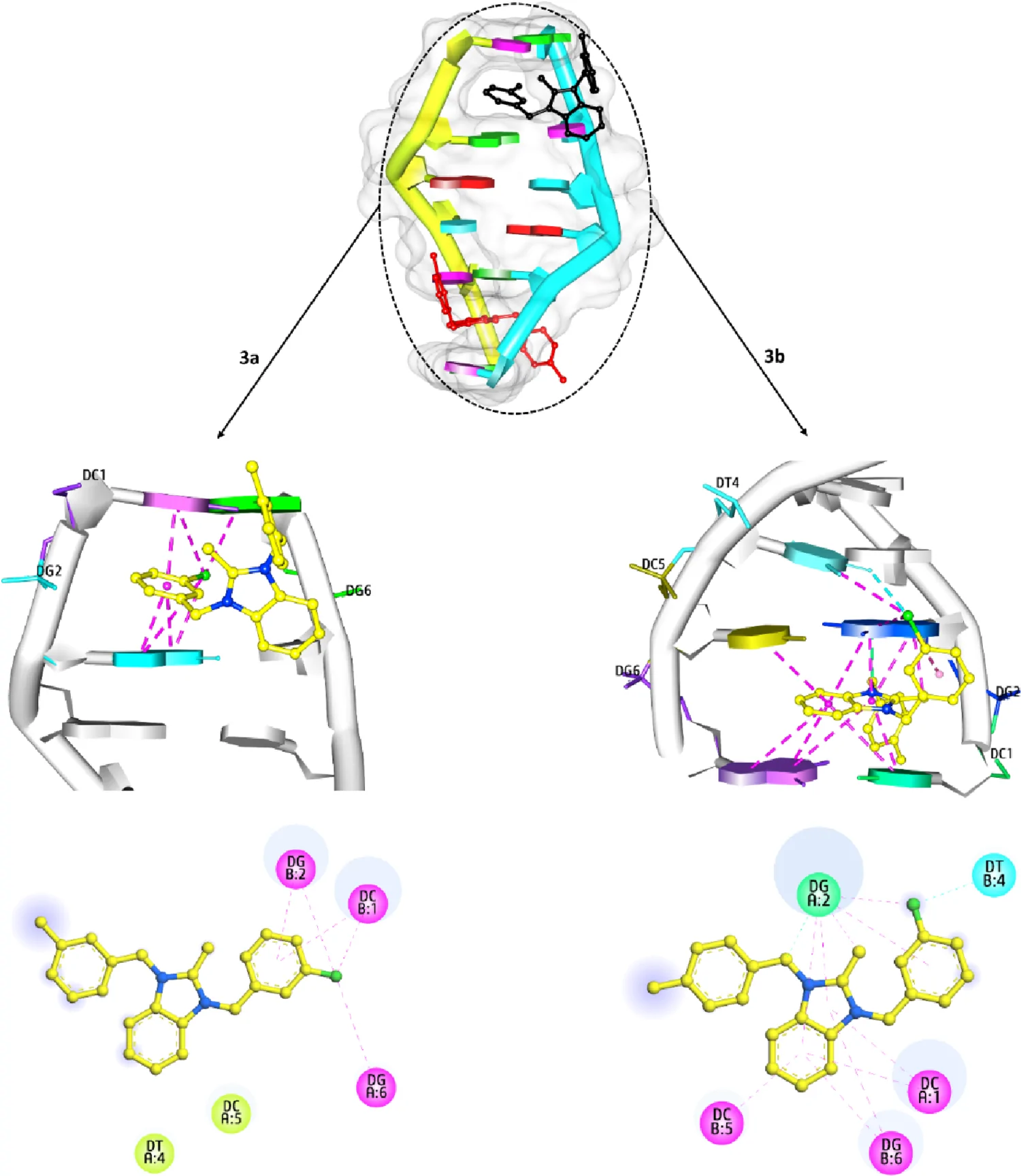
Docking finding of **3a** and **3b** in the binding with 1Z3F

To support the fluorescence spectroscopy results for DNA binding, which indicated that compound **3b** exhibits stronger affinity than **3a**, docking studies were conducted utilizing the DNA structure (PDB ID: 1Z3F). This study also allowed identification of the nucleotide base pairs involved in the interaction and the type of binding between the synthesized compounds and DNA. The calculated binding energies are −8.69 kcal/mol for **3a** and −8.90 kcal/mol for **3b**, showing good agreement with the trend observed experimentally in fluorescence measurements. Minor differences between theoretical and experimental values can be attributed to limitations of docking calculations, which do not fully account for solvent and buffer effects. The docking analysis further confirmed that **3b** binds slightly more strongly to DNA than **3a**, consistent with the biological and spectroscopic findings. Furthermore, docking demonstrates that the compounds 3a and 3b bind to DNA through an intercalation mode by locating some moiety between the DNA base pairs (Fig. 15). Compound **3a** forms hydrophobic interactions with DG2, DC1, and DG6 and van der Waals contacts with DT4 and DC5 base pairs. In contrast, compound **3b** exhibits hydrophobic interactions with DC5, DG6, DC1, and DG2, a hydrogen bond with DG2, and a halogen bond with DT4. Furthermore, hydrophobic interactions play key roles in stabilizing the complexes formed between the compounds and DNA, which confirms the presence of an intercalation mode between the **3a**/**3b** and DNA.

#### BSA interaction

**Fig. 16:**
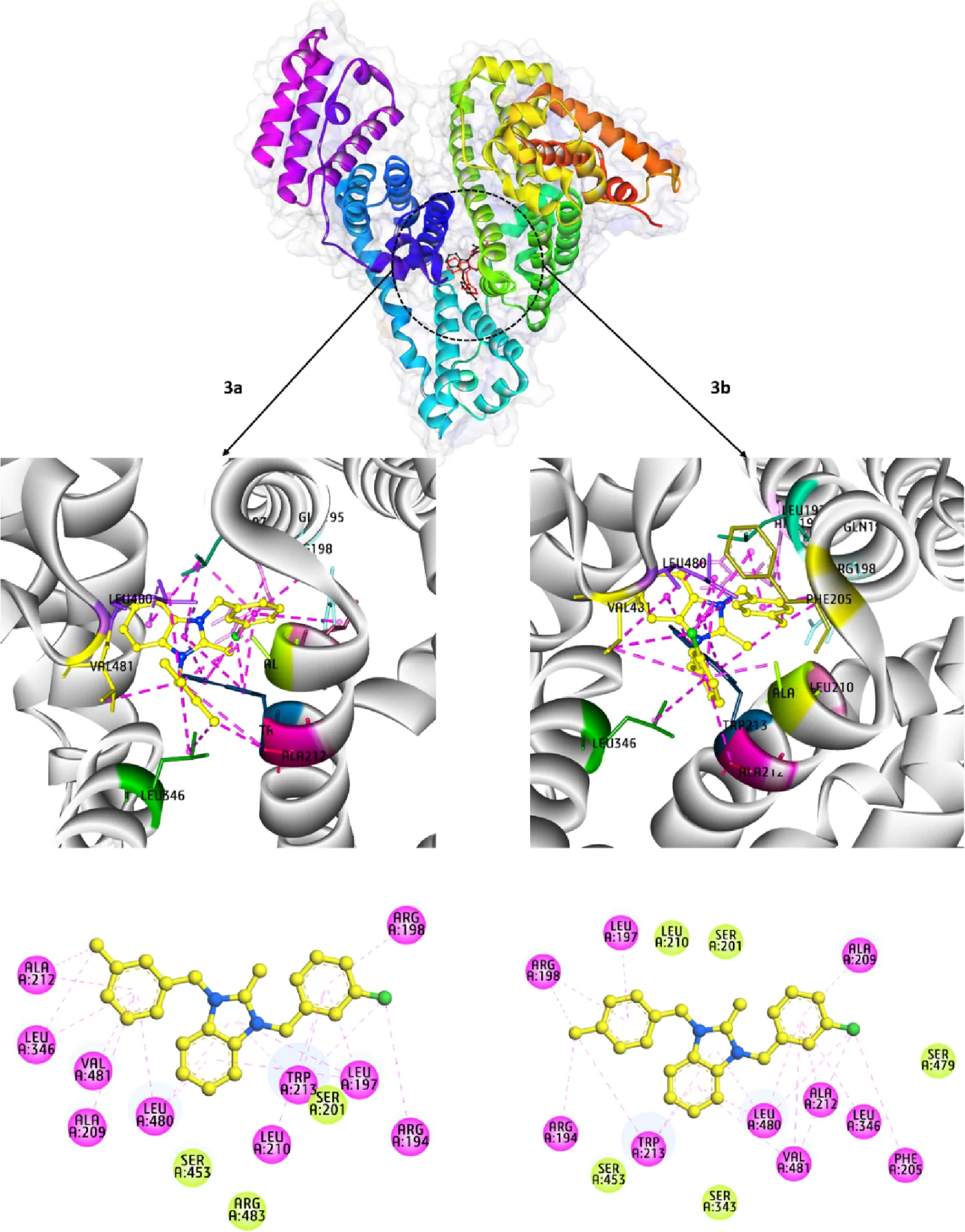
Docking finding of **3a** and **3b** in the binding with 4JK4

Because the interaction with BSA was experimentally examined using fluorescence spectroscopy, molecular docking simulations were additionally performed to visualize the binding mode and estimate binding affinity for compounds **3a** and **3b** toward BSA. The predicted docking conformations are presented in Fig. 16. The interaction analysis indicates that hydrophobic contacts represent the dominant stabilizing forces between the synthesized compounds and the protein cavity, accompanied by weaker van der Waals contributions. The calculated binding energies were −4.93 kcal/mol for **3a** and −5.10 kcal/mol for **3b**. These relatively small binding affinities are consistent with the experimental observations and are notably lower than those obtained for DNA binding. The weaker interaction with serum albumin suggests limited strong association with carrier proteins, which may be advantageous by reducing nonspecific protein binding and potential side effects related to extensive serum albumin sequestration.

## Conclusion

Recent advances in synthetic methodologies have markedly accelerated the discovery of novel compounds with potent anticancer activity directed toward specific molecular targets. These innovative strategies not only facilitate the efficient synthesis and structural optimization of complex molecules but also enable the design of compounds capable of modulating key regulatory pathways in cancer cells. The present study showed that the tested compounds have a significant anticancer potential against cancer cells. Specifically, these effects were demonstrated by modulating the key players of the apoptotic signaling and oxidative stress-regulating enzymes in cells through various pathways. These results also highlight the promising antitumor activity of the tested compounds. The interactions of compounds with CT-DNA and BSA were investigated utilizing fluorescence spectroscopy supported by DFT calculations and docking investigations. **3a** and **3b** exhibited strong binding affinity toward DNA, accompanied by static fluorescence quenching and an approximate 1:1 binding stoichiometry, suggesting an intercalative binding mode. In contrast, moderate binding affinity was observed with BSA, indicating limited nonspecific protein interaction. Computational studies revealed stable molecular geometries, favorable electronic properties, and significant noncovalent interactions consistent with experimental findings. Docking simulations further supported the spectroscopic results, demonstrating stronger binding of compound **3b** compared to **3a** toward DNA and biological targets. The combined experimental and theoretical results reveal the potential of these compounds as promising DNA-targeting candidates with relevant biological activity.

## Data Availability

Data will be made available on request.
